# Fisetin: An Integrated Approach to Identify a Strategy Promoting Osteogenesis

**DOI:** 10.3389/fphar.2022.890693

**Published:** 2022-05-16

**Authors:** Luca Dalle Carbonare, Jessica Bertacco, Salvatore Calogero Gaglio, Arianna Minoia, Mattia Cominacini, Samuele Cheri, Michela Deiana, Giulia Marchetto, Anna Bisognin, Alberto Gandini, Franco Antoniazzi, Massimiliano Perduca, Monica Mottes, Maria Teresa Valenti

**Affiliations:** ^1^ Department of Medicine, University of Verona, Verona, Italy; ^2^ Department of Neurosciences, Biomedicine and Movement Sciences, University of Verona, Verona, Italy; ^3^ Biocrystallography Lab, Department of Biotechnology, University of Verona, Verona, Italy; ^4^ Department of Surgery, Dentistry, Pediatrics and Gynecology, University of Verona, Verona, Italy

**Keywords:** osteogenesis, PLGA, fisetin, mesenchymal stem cells, differentiation

## Abstract

Flavonoids may modulate the bone formation process. Among flavonoids, fisetin is known to counteract tumor growth, osteoarthritis, and rheumatoid arthritis. In addition, fisetin prevents inflammation-induced bone loss. In order to evaluate its favorable use in osteogenesis, we assayed fisetin supplementation in both *in vitro* and *in vivo* models and gathered information on nanoparticle-mediated delivery of fisetin *in vitro* and in a microfluidic system. Real-time RT-PCR, Western blotting, and nanoparticle synthesis were performed to evaluate the effects of fisetin *in vitro*, in the zebrafish model, and in *ex vivo* samples. Our results demonstrated that fisetin at 2.5 µM concentration promotes bone formation *in vitro* and mineralization in the zebrafish model. In addition, we found that fisetin stimulates osteoblast maturation in cell cultures obtained from cleidocranial dysplasia patients. Remarkably, PLGA nanoparticles increased fisetin stability and, consequently, its stimulating effects on RUNX2 and its downstream gene SP7 expression. Therefore, our findings demonstrated the positive effects of fisetin on osteogenesis and suggest that patients affected by skeletal diseases, both of genetic and metabolic origins, may actually benefit from fisetin supplementation.

## 1 Introduction

Flavonoids are phenolic compounds commonly found in vegetables and fruits. Various flavonoids have been described, and it has been demonstrated that they produce biological effects through different mechanisms of action (Cederroth and Nef, 2009).

*In vitro* experiments have shown that flavonoids are able to modulate osteogenesis by affecting the physiology of bone-forming cells, that is, osteoblasts (Trzeciakiewicz et al., 2009).

It is well known that osteoblasts originate from mesenchymal progenitors through osteogenic differentiation (Kular et al., 2012). This process is regulated by different extracellular signals such as bone morphogenetic proteins, parathyroid hormone, Wnt, or hedgehog pathway (Soltanoff et al., 2009). Cellular signaling induces the expression of transcription factors, including Runt-related transcription factor 2 (Runx2) and osterix (SP7) (Marie, 2008). In particular, Runx2 is the osteogenic master gene, controlling proliferation and differentiation (Komori, 2010, 2). In humans, Runx2 gene mutations cause cleidocranial dysplasia (CCD, OMIM#119600), a skeletal disorder with aplasia/hypoplasia of clavicles and dental abnormalities (Lee et al., 1997). Runx2 transactivates downstream osteogenic genes such as osteocalcin, type I collagen alpha-1 (COL1A1 and COL1A2), or osteopontin (Ducy et al., 1997).

Among flavonoids, the polyphenol fisetin is found in vegetables and fruits such as strawberry, persimmon, and apple (Arai et al., 2000). *In vitro* experiments have demonstrated that fisetin can induce apoptosis in lung and prostate cancer cells (Sung et al., 2007; Khan et al., 2008a), it exerts antioxidant properties in retinal epithelial and neuronal cells (Ishige et al., 2001; Hanneken et al., 2006), and it has anti-inflammatory effects on macrophages and fibroblasts (Liu et al., 2010; Funakoshi-Tago et al., 2011). By testing fisetin *in vivo* models, it has been demonstrated that it can counteract tumor growth (Khan et al., 2008b), osteoarthritis (Zheng et al., 2017), and rheumatoid arthritis (Léotoing et al., 2013). Inflammation and diseases due to oxidative effects affect a large number of subjects; therapeutic molecules have an might impact on quality of life (De Franceschi et al., 2017). Recently, different molecular mechanisms have been proposed to support fisetin effects on osteogenesis, for example, GSK-3β phosphorylation and the consequent β-catenin activation (Molagoda et al., 2021) or the activation of the phosphoinositide-3-kinase/protein kinase B (PI3K-AKT) signaling pathway (Xu et al., 2021). In addition, it has been demonstrated that fisetin prevents inflammation-induced bone loss and promotes Runx2 upregulation in order to stimulate primary osteoblastic activity, thus improving the mineralization process (Léotoing et al., 2014). This last finding suggests that fisetin could be an exploitable tool for counteracting skeletal osteopenia. Interestingly, nanoencapsulation by using nanoemulsion and liposomal formulations as well as polymeric nanoparticles (NPs), based on poly(ε-caprolactone) (PCL) (Kadari et al., 2017) or poly(D, L-lactic-co-glycolic acid)-block-poly(ethylene glycol) carboxylic acid (PLGA-PEG-COOH) (Sechi et al., 2016) have been proposed to improve fisetin supplementation.

However, an integrated approach aiming to achieve fisetin employment in osteogenesis promotion has not been undertaken so far. Therefore, in order to evaluate its effectiveness in counteracting bone diseases, we assayed fisetin supplementation both *in vitro* and in the *Danio rerio in vivo* model. We also assayed the effects of fisetin in the *ex vivo* model of cleidocranial dysplasia (CCD), a dominantly inherited disease affecting osteogenic differentiation. In addition, we generated a nanoparticle complex with elevated drug loading [PLGA (Fis)]. By using a microfluidic system, we assayed the ability of [PLGA (Fis)] to cross the human intestinal epithelial tissue.

## 2 Materials and Methods

### 2.1 XTT Test

The Cell Proliferation Kit II (XTT Chemicon) was used to evaluate cell viability, as previously described (Deiana et al., 2019, 6). Six replicates in three independent experiments were tested.

### 2.2 Cell Cultures

Human mesenchymal stem cells (hMSC, PromoCell, Heidelberg, Germany) were used by culturing at a density of 5 × 10^4^ cells with the mesenchymal stem cell growth medium (PromoCell) or osteogenic differentiation medium (PromoCell, Heidelberg, Germany) and incubated at 37°C in a humidified atmosphere with 5% CO_2_. Differentiating cells were then used for further analyses. Human dermal fibroblast cultures were established from explanted skin biopsies taken from patient P1 [mutation: c.897T>G->p (Tyr299*), male, 8 years old], P2 [c.1019del-> p (Ser340*), female, 10 years old], and a healthy age-matched control with appropriate consent, as previously reported (Venturi et al., 2012). The cells were cultured with high-glucose DMEM (ECB7501L, EuroClone, Milano, Italy) supplemented with 10% FBS (10270-106, Gibco, Life Technologies Limited, Paisley, United Kingdom), 2 mM l-glutamine (5-10K00-H, BioConcept AG, Paradiesrain, Allschwil, Switzerland), 100 U/ml penicillin, and 100 μg/ml streptomycin (penicillin–streptomycin; ECB3001D, EuroClone, Milano, Italy), as previously described (Venturi et al., 2012).

### 2.3 Zebrafish

The zebrafish experiments were performed at the CIRSAL (Interdepartmental Centre of Experimental Research Service) of the University of Verona, Italy, under ethical authorization n. 662/2019-PR of 16/09/2019.

The embryos were obtained with *nacre* adults (ZFIN database ID: ZDB-ALT-990423-22), according to standard procedures (Kimmel et al., 1995; Whitlock and Westerfield, 2000). Preliminary tests at 2.5, 5, and 10 µM had shown no effect on the modulation of osteogenic gene expression. Thus, the embryos were grown at 33°C in water containing 15 µM fisetin from 2 days post-fertilization (dpf). The zebrafish embryos were supplemented with fisetin up to 1 week (experimental endpoint, 9 dpf). At the end of the treatment, the zebrafish embryos were euthanized and collected for performing molecular analyses were performed, as described later. To evaluate bone formation, we performed calcein staining, as previously reported (Du et al., 2001). Then, imaging was performed by using a Leica M205FA fluorescence microscope (Leica Microsystems, Wetzlar, Germany). The stained areas were quantified with ImageJ software, as previously reported (Du et al., 2001).

Adult zebrafish (15–20 months) were grown in water in the presence of 15 µM fisetin for 14 days (for 1 week of supplementation, no effect on the modulation of osteogenic gene expression was observed). At the experimental endpoint, the adult zebrafish were euthanized and collected for the staining procedures and molecular analyses, as reported later.

### 2.4 Total RNA Extraction and Reverse Transcription

Pellets from cells or zebrafish samples were collected and stored at −80°C until use. The samples were processed for RNA extraction by using an “RNeasy^®^ protect mini kit” (Qiagen, Hilden, Germany), as previously reported (Cecconi et al., 2019). The RNA samples were quantified using a Qubit™ RNA HS assay kit” (Invitrogen, Carlsbad, United States) and a Qubit 3 Fluorometer (Invitrogen by Thermo Fisher Scientific, REF Q3321). RNA (1 mg) was reverse-transcribed using the first-strand cDNA synthesis kit (GE Healthcare, Little Chalfont), as per the manufacturer’s instruction, and a SimpliAmp Thermal Cycler (Applied Biosystems by Thermo Fisher Scientific, REF A24812). RNA and cDNA samples were stored at −80°C until use.

### 2.5 Real-Time PCR

To investigate gene expression modulation, we performed real-time PCR analyses as reported previously (Dalle Carbonare et al., 2019).

Briefly, predesigned, gene-specific primers and probe sets for each gene [RUNX2, hs00231692_m1; OSTERIX (SP7), hs00541729_m1; collagen, type i, alpha 2 (COL1A2), hs01028956_m1; osteonectin (SPARC), hs00234160_m1; OSTEOPONTIN (SPP1), hs00167093_m1, B2M, hs999999_m1 (housekeeping); and GAPDH, 0802021 (housekeeping)] were obtained from assay-on-demand gene expression products (Thermo Fisher Corporation, Waltham, MA, United States). In addition, the following custom primer sets (Invitrogen, Carlsbad, CA, United States) were also used: runx2a (fw GAC​GGT​GGT​GAC​GGT​AAT​GG, rv TGC​GGT​GGG​TTC​GTG​AAT​A), runx2b (fw CGG​CTC​CTA​CCA​GTT​CTC​CA, rv CCA​TCT​CCC​TCC​ACT​CCT​CC), rank (fw GCA​CGG​TTA​TTG​TTG​TTA, rv TAT​TCA​GAG​GTG​GTG​TTA​T), and housekeeping gene actb1 (fw CCC​AAA​GCC​AAC​AGA​GAG​AA, rv ACC​AGA​AGC​GTA​CAG​AGA​GA). Ct values for each reaction were determined using TaqMan SDS analysis software (Applied Biosystems; Foster City, California, United States) as reported previously. To calculate relative gene expression levels between different samples, we performed the analyses by using the 2−ΔΔCT method as previously reported (Dalle Carbonare et al., 2019).

### 2.6 Cellular Reactive Oxygen Species Detection

We measured ROS levels by staining cells with the DCFDA cellular ROS detection assay kit (Abcam), as previously reported (Deiana et al., 2019). After the staining, the cells were analyzed by measuring the produced fluorescence (ex/em = 485/535 nm) in the endpoint at the VictorX4 instrument (Perkin Elmer, Milan, Italy) according to the manufacturer’s protocol.

### 2.7 Western Blotting

RUNX2 protein levels were separated by using SDS-PAGE and investigated by using Western blot analyses as previously reported (Brugnara and De Franceschi, 1993). Briefly, proteins were extracted by using a RIPA buffer (Thermo Fisher Scientific, Waltham, MA, United States), and the proteins were quantified by BCA assay (Thermo Fisher Scientific, Waltham, MA, United States). The proteins were separated by sodium dodecyl sulfate–polyacrylamide gel electrophoresis (SDS-PAGE) and then were transferred onto polyvinylidene difluoride (PVDF) membranes (Thermo Fisher Scientific, Waltham, MA, United States). The PVDF membranes were then probed with the primary antibody for RUNX2 (Cell Signaling, 8486) and β-actin (BA3R) (Thermo Scientific), and secondary antibodies anti-mouse (Cell Signaling, 7076) and anti-rabbit (Cell Signaling 7074). Signals were detected using a chemiluminescence reagent (ECL, Millipore, Burlington, MA, United States), as previously reported (de Franceschi et al., 2004). Images were acquired by an LAS4000 Digital Image Scanning System (GE Healthcare, Little Chalfont, United Kingdom). The densitometric analysis was performed by ImageQuant software (GE Healthcare, Little Chalfont, United Kingdom). Protein optical density was normalized to β-actin.

### 2.8 Alizarin Red Staining *In Vitro* Experiments

Calcium deposition in differentiating osteogenic cells was evaluated by Alizarin Red staining, as previously described (Dalle Carbonare et al., 2019). Briefly, after 21 days of culture in the differentiating medium, the cells were fixed with 70% ethanol and washed with water. The cells were then stained with 40 mM Alizarin Red S for 5 min at pH 4.1 and rinsed for 15 min. The stained area was quantified with ImageJ software (NIH, Bethesda, MD, United States), as previously reported (Dalle Carbonare et al., 2019). Six independent experiments were performed.

### 2.9 Zebrafish Staining

Calcein staining in zebrafish larvae was performed, as previously reported (Du, Frenkel et al., 2001). The imaging was performed using a Leica M205FA fluorescence microscope (Leica Microsystems, Wetzlar, Germany). The stained area was quantified by using ImageJ software, as previously reported (Du et al., 2001).

Bone and cartilage staining in adult zebrafish was performed as described in Sakata-Haga et al. (2018). Briefly, the euthanized fish were immersed into the fixative solution (5% formalin, 5% Triton X-100, 1% potassium hydroxide (KOH) and gently rocked for 48 h  at room temperature (RT). Then, we proceeded either to the cartilage staining step for double staining or directly to the bone staining step for bone-only staining. For cartilage staining, the specimens were immersed into C-Staining Solution (70% ethanol, 20% acetate, 0.015–0.02% Alcian Blue)” overnight at 20°C, then washed in C-Staining medium (containing 1% Triton X-100) overnight at 20°C and washed with 50–70% ethanol. Then the protocol proceeded to the bone staining step. The specimens were immersed in B-Staining medium (20% ethylene glycol and 1% KOH) and then in “B-Staining solution (0.05% Alizarin Red S, 20% ethylene glycol, 1% KOH) overnight at 20°C. The specimens were then washed with clearing solution (20% Tween 20, 1% KOH) while gentle rocking for 12 h, and the stocking was performed in glycerol 100%. The stained area was quantified by using ImageJ software, as previously reported (Du et al., 2001).

### 2.10 Fisetin Stability Analysis

Tests were carried out in order to evaluate the stability of fisetin in a complete culture medium (DMEM with 10% FBS and 1% PEN/STREP) at different experimental times (time = 0 vs. 8 h). Starting from a 10 mM stock solution of fisetin in DMSO, we obtained a 100 uM solution, which was analyzed by HPLC at time T_0_ and after 6 h. In particular, the measurements in HPLC were performed at time T_0_ and after 30 min, 1, 2, 4, and 6 h. The solution was kept at 37°C and away from direct light to simulate experimental conditions.

### 2.11 PLGA Nanoparticles Synthesis

PLGA [poly(DL-lactide-co-glycolide), 50:50 lactide-to-glycolide ratio, CAS 26780-50-7], PVA [poly(vinyl alcohol), CAS 9002-89-5], and acetone (≥99% purity, 1.00013) were purchased from Merck. Fluorescein isothiocyanate (FITC, CAS 27072), cellulose membrane dialysis tubing (CASD9777-100), dimethyl sulfoxide (DMSO, ≥ 99% purity D-5879) were purchased from Sigma-Aldrich. Fisetin was purchased from Santa Cruz Biotechnology (SC-276440).

The protocol used for the production of PLGA nanoparticles loading fisetin [PLGA (Fis)] is based on a single emulsion evaporation method, under sterile conditions at 20°C (Makadia and Siegel, 2011; Gaglio et al., 2019). A measure of 20 mg of the polymer and 4 mM (1.14 mg) of fisetin were co-dissolved in 1 ml of DMSO 100%; the obtained organic phase was added dropwise under stirring (2,000 RPM) to 10 ml of 0.5% polyvinyl alcohol (PVA) aqueous solution and left overnight to evaporate the organic phase. Afterward, the preparation was pelleted at 4°C 11,000 rpm for 20 min (Eppendorf Centrifuge 5804R), and the nanoparticles were collected and washed twice with 10 ml of Milli-Q water. Finally, the purified nanoparticles were resuspended in 1 ml of phosphate-buffered saline (PBS) solution pH 7.4 (or NaCl 0.9%) for the subsequent analysis and storage at 4°C, otherwise freeze-dried. Empty nanoparticles were prepared with the same protocol avoiding the addition of fisetin to the reaction. Furthermore, to perform an internalization study and visualize nanoparticles inside cells, PLGA NPs co-delivering fisetin and fluorescein isothiocyanate (FITC) [PLGA (Fis and Fitc)] were prepared by dissolving 20 mg of PLGA in a mixture of 640 ul of acetone and 360 ul of fisetin/FITC DMSO solution; the molar ratio between fisetin and FITC is 1:1. The other steps were the same as described previously.

### 2.12 Size and ζ-Potential Characterization

#### 2.12.1 Dynamic Light Scattering and ζ-Potential

The size and ζ-potential of PLGA nanoparticles were estimated at 25°C by dynamic light scattering (DLS) (Nano Zeta Sizer ZS, ZEN3600, Malvern Instruments, Malvern, Worcestershire, United Kingdom). Nanoparticles were resuspended in PBS, used as a stock suspension, and were diluted 20 times in PBS for size determination and in 10 mM NaClO_4_ pH 7.5 for ζ-potential measurements; data were collected in triplicate and analyzed by ZetaSizer software.

#### 2.12.2 Nanosight Tracking Analysis

To support DLS data, a Nanosight tracking analysis was performed on PLGA (Fis) and empty NPs (Malvern NanoSight NS300). Due to the high concentration, each sample was diluted 10,000 or 5,000 times; 1,498 frames divided into 3 runs of 60 s were recorded at a camera level of 13, and the analysis was performed with a detection threshold in the range 5–7. Finally, the number of particles/ml was estimated as well.

#### 2.12.3 Atomic Force Microscopy

A volume of 20 μl of each sample (prepared as in mentioned the previous section) was loaded on a bracket covered by an inert mica surface. After 15 min of solvent evaporation, the analysis was performed using an NT-MDT Solver Pro atomic force microscope with NT-MDT NSG01 golden coated silicon tip in semi-contact mode with different scanning frequencies (3–1 Hz) in order to produce optimized AFM images. The microscope was calibrated by a calibration grating (TGQ1 from NT-MDT) in order to reduce nonlinearity and hysteresis in the measurements. Finally, images were processed with the Scanning Probe Image Processor (SPIP™) program (Friis Jan, 2009), and a statistical study was performed to compare results to DLS and Nanosight data.

### 2.13 Spectroscopy, Encapsulation Efficiency and Release Study

#### 2.13.1 Absorbance and Emission Spectroscopy

The absorbance pattern of each sample resuspended in PBS or water was analyzed using a Thermo Fisher Evolution 201 UV-Visible Spectrophotometer in the range 250–600 nm to assess the presence of fisetin inside nanoparticles and the co-presence of fisetin and FITC. Moreover, the emission pattern was recorded upon excitation at 360 and 495 nm (excitation wavelengths for fisetin and FITC, respectively) by using a Jasco Spectrofluorometer FP-8200.

#### 2.13.2 Encapsulation Efficiency and Drug Loading

To quantify the amount of fisetin entrapped, PLGA (Fis) NPs were dissolved in DMSO and analyzed using the calibration line (Fig x). Encapsulation efficiency (EE) and drug loading (DL) were estimated using the following equations:

$$EE\;\left(\%\right)=\;\frac{Fisetin_{loaded}}{Fiseten_{fed}}\;\times \;100,$$

$$DL\left(\%\right)=\;\frac{mg\;of\;Fisetin_{loaded}}{mg\;of\;PLGA}\;\times \;100.$$

### 2.14 Stability and *In Vitro* Drug Release

To assess the capacity of the nanoparticles to retain entrapped fisetin over time, a first release study was performed in a total volume of 1 ml, at different temperatures (4 and 37°C) and different media (water, PBS NaCl 0.9%, and citric acid pH 5). Dialysis was used to carry out the *in vitro* drug release studies increasing the final volume. PLGA (Fis) NPs were introduced into the dialysis bag (14,000 da molecular weight cutoff, Sigma Aldrich, D9777-100FT) and placed in 100 ml of PBS pH 7.4 containing 0.1%v/v Tween 80 as the release media and stirred at 100 rpm. Samples were collected at different time intervals and replaced with an equal volume of media to maintain the sink condition. The released fisetin was quantified using a calibration curve obtained by UV–visible spectroscopy at 360 nm (Kadari et al., 2017).

### 2.15 Fluid-Dynamic Intestinal Model Resembling Systemic Administration for PLGA (fis)

A compartmental fluidic device (commercialized as MIVO React4life S.r.l., IT) was used to perform *in vitro* drug efficacy tests. The 3D fluidic model was performed as follows: 1) 24-well size inserts containing human intestinal tissue (EpiIntestinal by Mattek) were placed and cultured within the device, forming two fluidically independent chambers: the donor and the receiver; 2) both chambers were filled with the culture medium; and 3) the receiver chamber was connected to the peristaltic pump to form a closed-loop fluidic circuit containing 3.8 ml medium circulating at a rate of 0.3 cm/s, to simulate the capillary flow rate. Therefore, a medium containing PLGA (fis) was added to the donor chamber.

### 2.16 Statistical Analysis

Results were expressed as mean ± SD. The statistical analysis was assessed by Student’s paired t-test comparing each treatment to the control. Differences were considered positive when *p* < 0.05. For *in vitro* data, analyses were applied to experiments carried out at least three times. We used SPSS for Windows, version 22.0 (SPSS Inc., Chicago, IL, United States), to analyze the data.

## 3 Results

### 3.1 Fisetin Counteracts ROS Levels During Osteogenic Differentiation

**FIGURE 1:**
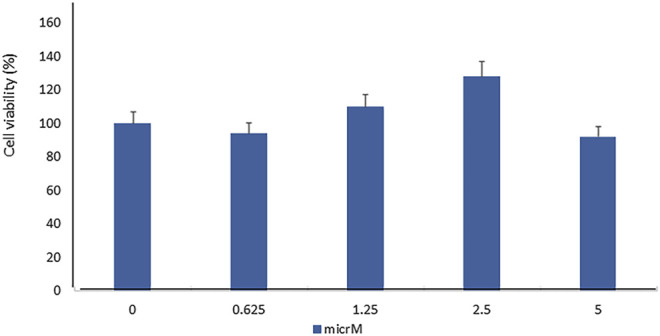
Fisetin supplementation did not affect MSC viability in a statistically significant manner. The cell viability was evaluated by performing the XTT test in hMSCs cultured for 7 days in the presence of fisetin ranging from 0 to 5 µM.

In order to evaluate the toxic effect of fisetin, we assayed the viability of mesenchymal stem cells treated with fisetin for 24 h at concentrations ranging from 0 to 5 µM. As shown in Figure 1, fisetin did not affect cell viability at any tested concentration.

**FIGURE 2:**
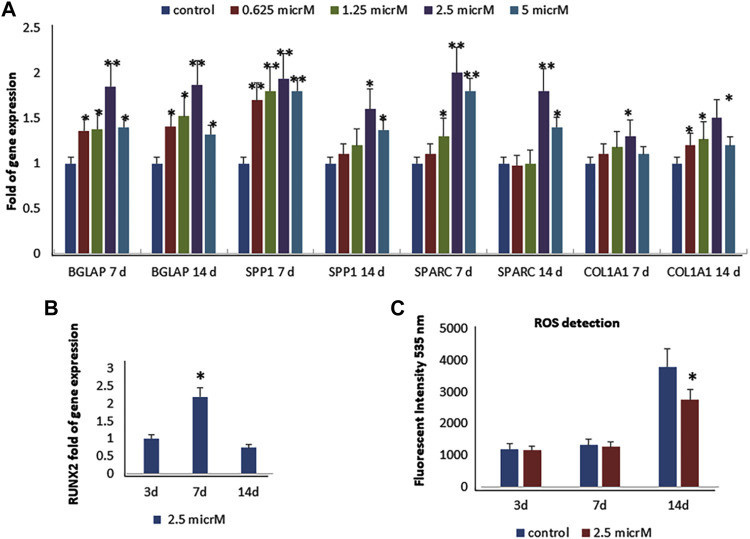
Effects of fisetin supplementation during osteogenic differentiation. The upregulation of osteogenic genes was much more evident when fisetin was supplemented at a concentration of 2.5 µM **(A)**. Expression of RUNX2 in treated cells decreased after 7 days of differentiation **(B)**. ROS levels were modulated by fisetin supplementation at the late differentiation phase (14 days of differentiation) **(C)**. Analyses were performed in hMSCs cultured in the presence of the osteogenic differentiation medium. *p < 0.05; **p < 0.01; ***p < 0.005.

As previously reported (Molagoda et al., 2021), we observed that fisetin promotes osteogenic differentiation (Supplementary Figure S1); the upregulation of osteogenic genes (BGLAP, SPP1, SPARC, and COL1A1) was much more evident when fisetin was supplemented at a concentration of 2.5 µM in MSCs (Figure 2A). RUNX2 expression in treated cells exerted the same modulation generally observed during osteogenic differentiation. In fact, RUNX2 upregulation was observed until after 7 days (Figure 2B) (Ardeshirylajimi et al., 2014). In order to assay fisetin’s effects on oxidative stress, which affects osteogenic modulation (Atashi et al., 2015), we measured ROS levels during differentiation. As shown in Figure 2, we observed reduced ROS levels in fisetin-treated cells during the late phase of differentiation (C).

**FIGURE 3:**
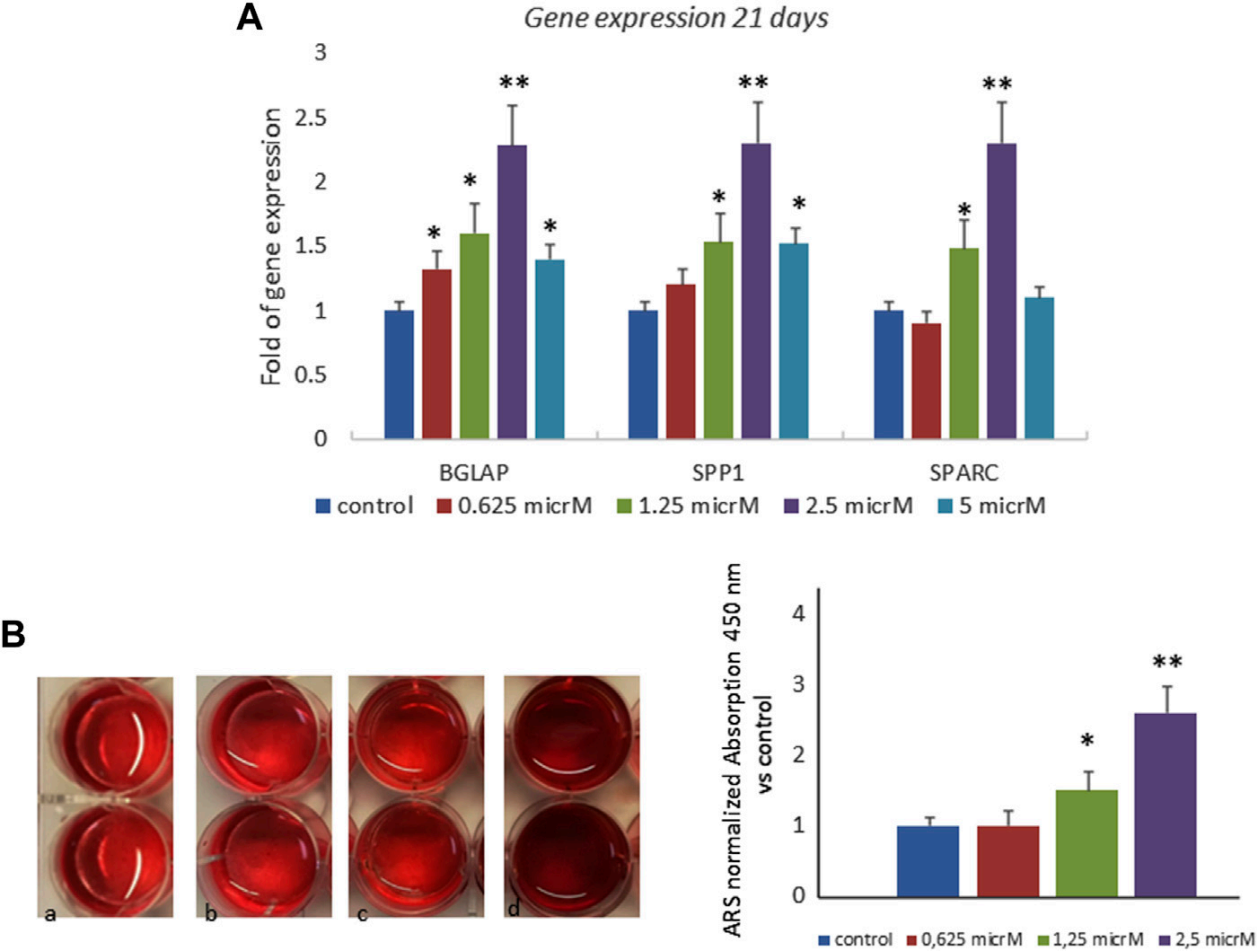
Effects of fisetin supplementation on the late phase of osteogenic differentiation. Fisetin increased the expression levels of osteogenic genes **(A)** and the mineral deposition **(B)** after 21 days of differentiation (mineralization phase) (a, control; b, 0.625 µM; c, 1.25 µM; d, 2.5 µM) *p < 0.05; **p < 0.001. Magnification ×4.

We also investigated the effects of fisetin in the late phase of osteogenic differentiation, and we cultured MSCs in an osteogenic medium with or without fisetin for 21 days. The effects of 2.5 µM fisetin supplementation were also observed during the late phase of osteogenic maturation of MSCs. As shown in Figure 3, fisetin supplementation increased the expression levels of RUNX2 downstream genes related to the late osteogenic phase (A) and to mineral deposition (B).

### 3.2 Fisetin and Pro-Osteogenic Molecules

**FIGURE 4:**
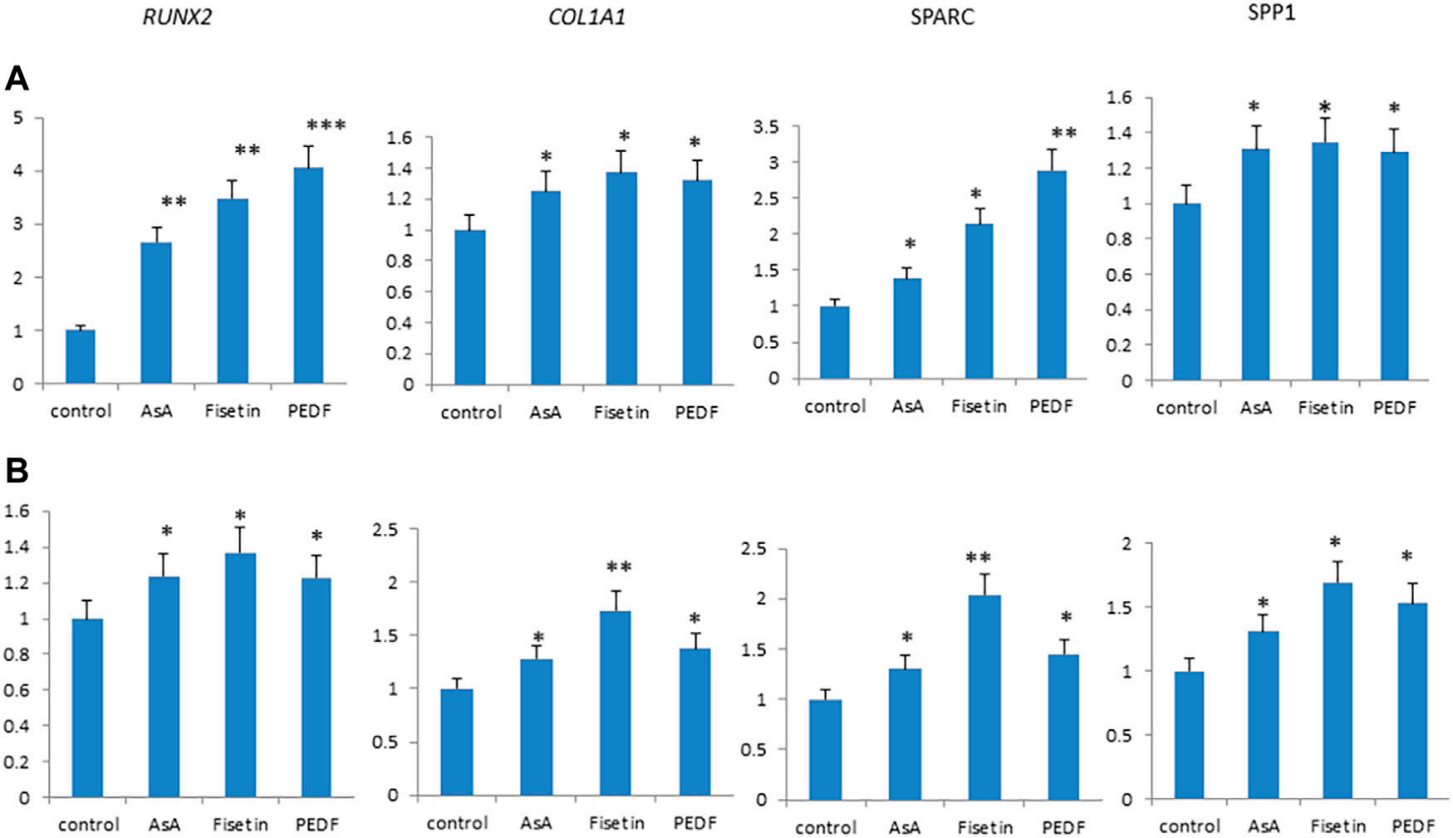
Fisetin, PEDF and AsA were able to upregulate osteogenic genes expression in MSCs after 7 **(A)** and 14 **(B)** days of supplementation. *p < 0.05; **p < 0.01; ***p < 0.005.

Fisetin effects were then compared with those of other supplemental molecules known to improve bone tissue quality. In particular, we compared the effects of fisetin, ascorbic acid, and pigment epithelium-derived factor (PEDF) supplementation, respectively, on MSC osteogenic differentiation. As shown in Figure 4, fisetin induced the upregulation of all tested osteogenic genes after 7 (A) and 14 (B) days of differentiation. Gene modulation induced by fisetin treatment was similar to that observed by treating cells with PEDF and AsA. COL1A1 and SPARC gene expressions induced by fisetin after 14 days of differentiation were more pronounced than PEDF and AsA treatment.

### 3.3 Fisetin Increases Osteogenic Differentiation and Mineralization in Larvae and Adult Zebrafish Model

To evaluate the effects of fisetin *in vivo*, we used the *Danio rerio* (zebrafish) model. In particular, zebrafish larvae were grown in water at 33°C after 2 days post-fertilization (dpf) in the presence/absence of fisetin. Preliminary tests at 2.5, 5 and 10 µM showed no effect on the expression of osteogenic genes compared to untreated zebrafish. However, we observed increased osteogenic gene expression in zebrafish treated with 15 µM fisetin. Thereafter, we used 15 µM concentration in all experiments.

**FIGURE 5:**
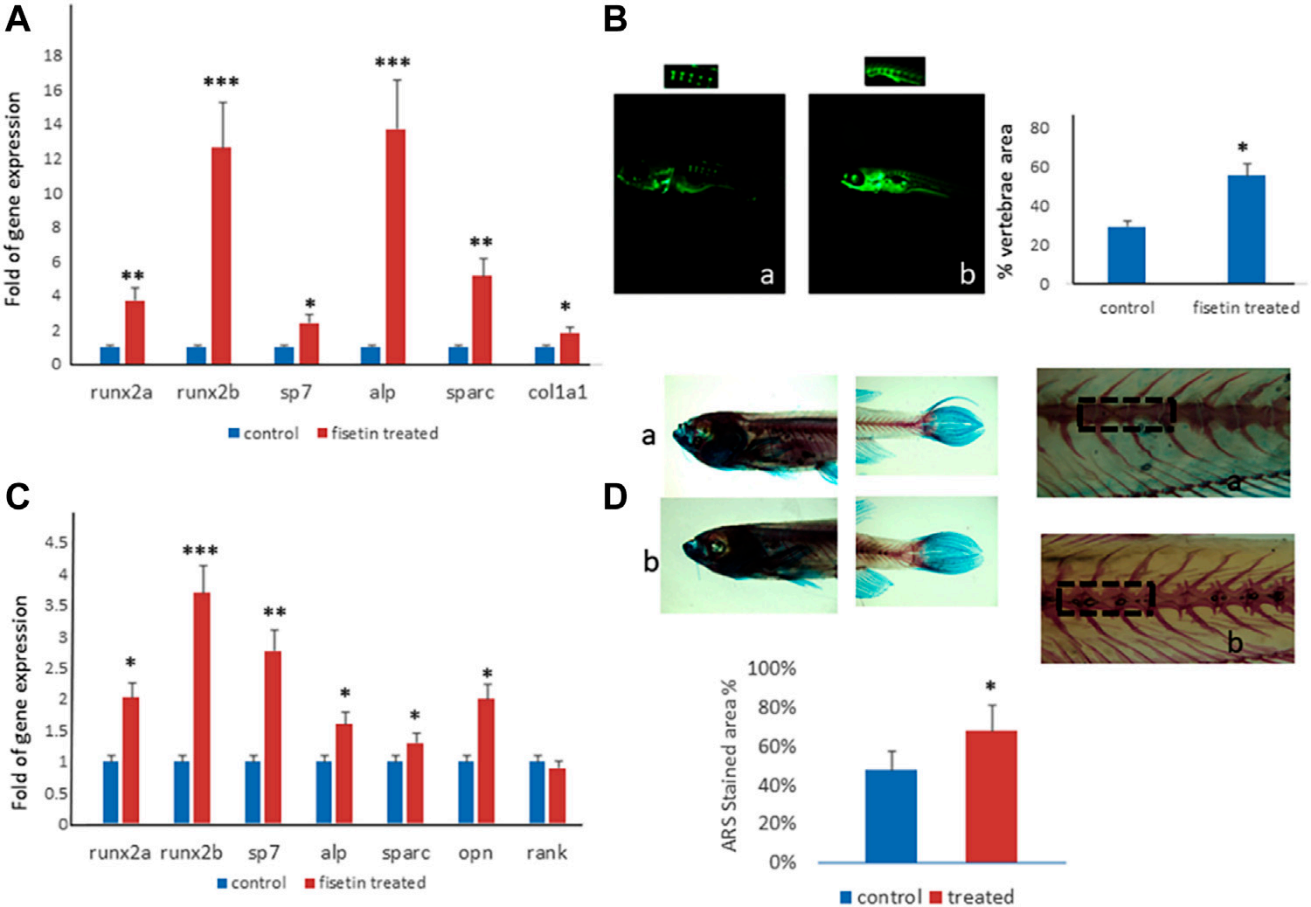
Fisetin effects on in vivo model. Fisetin supplementation increased osteogenic genes expression in zebrafish larvae after 7 (9 dpf) days of treatment **(A)**. The fluorescence density produced by calcein staining showed an increased vertebrae area in larvae after 7 days of fisetin **(B)**. Osteogenic genes were upregulated also in adult zebrafish supplemented with fisetin for 14 days **(C).** Fisetin supplementation for 14 days increased bone mineralization, evaluated by Alizarin red staining (ARS) in adult zebrafish **(D)**. *p<0.05; **p<0.01; ***p<0.005.

After 7 (9 dpf) days of treatment, zebrafish were euthanized and collected to perform gene expression analyses or staining. As shown in Figure 5A, the upregulation of osteogenic genes in fisetin-supplemented zebrafish larvae could be observed.

In order to record the direct effects of fisetin on bone mineralization, we quantified the vertebrae area evaluated by calcein staining. We observed calcein fluorescence density under microscopic inspection, and we analyzed fluorescence levels by using digital methods. Calcein staining showed an increased vertebrae area in larvae after 7 days of fisetin treatment, compared to untreated larvae (Figure 5B).

Interestingly, the upregulation of osteogenic genes was also observed in adult zebrafish (15–20 months) supplemented with fisetin for 14 days (Figure 5C). In addition, we also observed increased bone mineralization, evaluated by Alizarin Red staining (ARS) in adult zebrafish treated with fisetin for 14 days. In fact, in adult zebrafish, more intense ARS and a higher percentage of positive ARS areas were observed in fisetin-treated zebrafish than in untreated zebrafish (Figure 5D).

### 3.4 Fisetin Improves Osteogenic Maturation in Cells of Pediatric Patients Carrying RUNX2 Mutations

**FIGURE 6:**
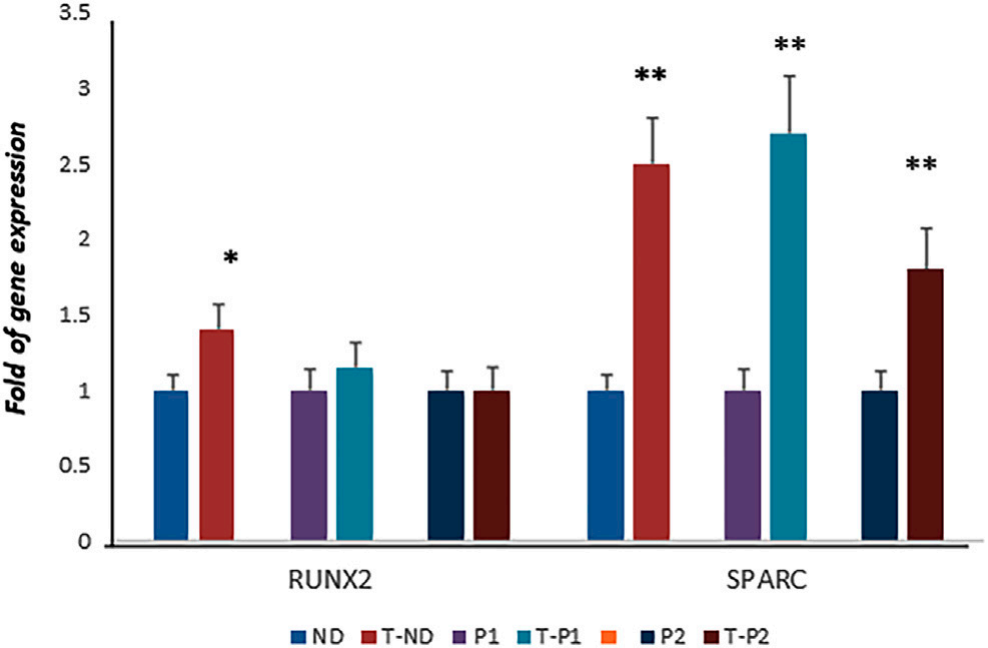
RUNX2 expression was not affected by fisetin in CCD patients. However, fisetin supplementation strongly upregulated the expression of SPARC in control as well as in osteogenic differentiating cells of both RUNX2 mutant patients (normal control vs treated normal control, untreated patients' cells vs treated patients' cells respectively)) (D). *p*<0.05; **p<0.005. ND, Normal Donor; T-ND, Treated Normal Donor; P1, Patient 1; T-P1, Treated Patient 1; P2, Patient 2; T-P2, Treated Patient 2.

Considering the positive effects of fisetin on osteogenic differentiation, we speculated about its possible beneficial effects in the context of skeletal pathologies. Therefore, we investigated the effects of fisetin in *ex vivo* models of cleidocranial dysplasia (CCD, OMIM#119600), a dominantly inherited skeletal disease. In particular, we obtained fibroblast-like cells from two unrelated pediatric patients affected by CCD carrying mutations in exon 7 of one RUNX2 allele, as we previously reported (Dalle Carbonare et al., 2021). In our previous study, we reported that RUNX2 gene expression levels in both patients were similar to those in the normal control, whereas SPARC gene expression was lower in both patients than in the control (Dalle Carbonare et al., 2021). However, when the cells were cultured in the presence of fisetin, we observed SPARC upregulation in patient’s cells (compared to untreated patients’ cells), whereas RUNX2 levels remained unchanged, suggesting fisetin’s ability to restore osteogenic maturation in this *ex vivo* skeletal disorder model (Figure 6).

### 3.5 Fisetin-Embedded Nanoparticles [PLGA (Fis)] Prevent Fisetin Degradation

**FIGURE 7:**
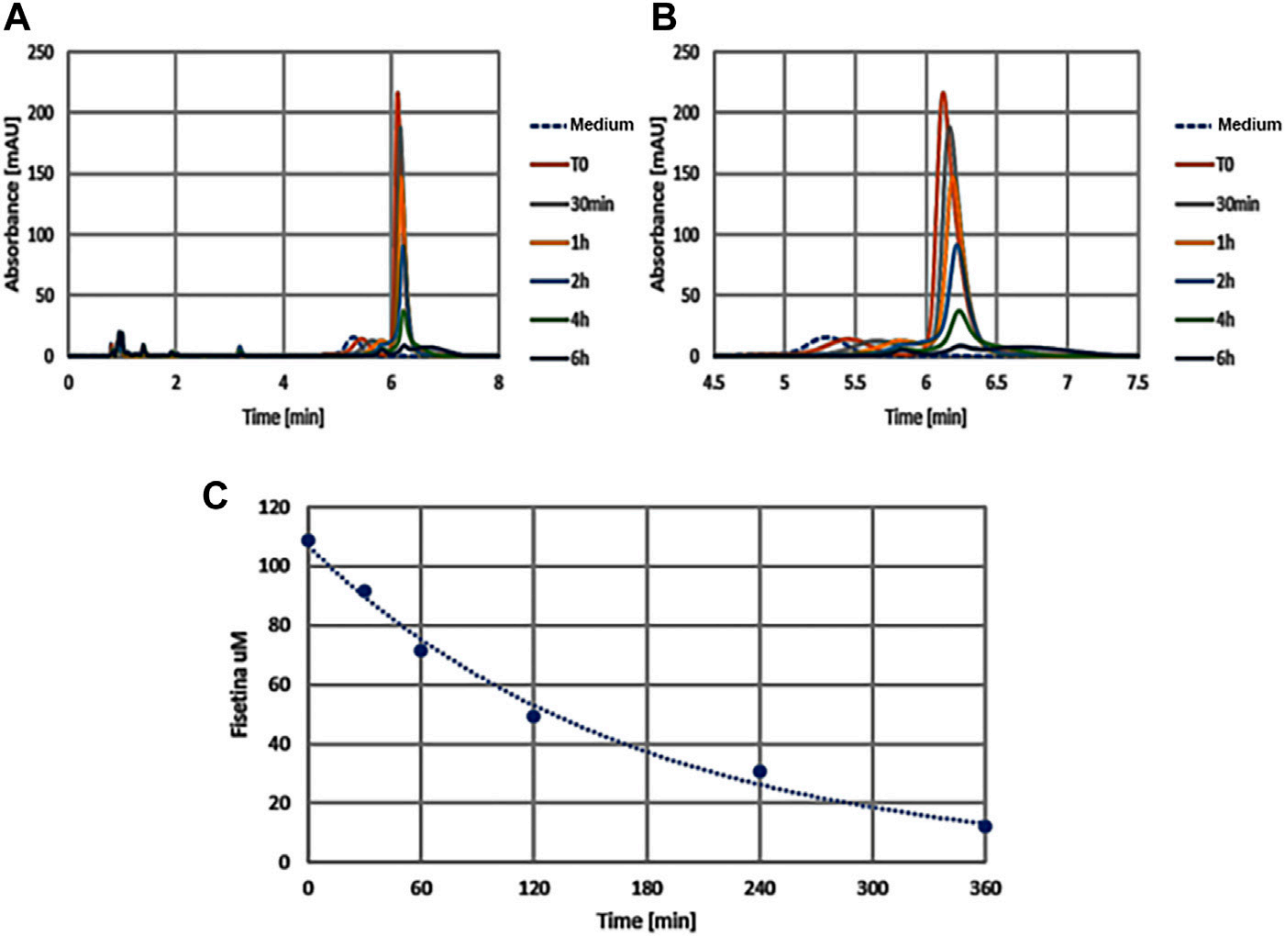
The graphics show absorption peaks of fisetin at 360 nm in culture medium at different times (in x axis): with the passage of time the molecule degrades and the peak is lowered, until it disappears later 6h. The image on the left **(A)** shows the entire duration of the run (from 0 to 8h time lapse in culture medium), while the one on the right **(B)** only around the retention time (from 4.5 to 7h). As control we used the colture medium (Medium) without fisetin supplementation. The degradation kinetics of fisetin is shown in graphic **C**.

With the aim of providing an efficient fisetin supplementation for the improvement of bone quality, we tested the characteristics of this molecule. In order to verify fisetin’s stability, we analyzed its concentration in a culture medium at different time points. As shown in Figure 7, we observed fisetin degradation upon the observation time lapse. In particular, fisetin was almost completely degraded after 6 h. Consequently, we generated fisetin containing PLGA particles [PLGA (Fis)] to prevent its degradation in order to make the supplementation available for human use. We compared our nanoformulation with data of fisetin encapsulation into PLGA previously published by Sechi et al., Kadari et al., and Liu et al. (Table 1) (Sechi et al., 2016; Kadari et al., 2017; Liu et al., 2021).

**TABLE 1:** Particle size, polydispersity index (PDI), encapsulation efficiency (EE), and drug loading (DL) data for present nanoparticles [PLGA (Fis)*] compared with those of Kadari et al** Sechi et al *** and Liu et al ****. *Measurements were performed in triplicate.

Nanoformulation	Particle size (NM)	PDI	ζ-Potential (MV)	EE (%)	DL (%)
PLGA (Fis)*	139.2 ± 4.0	0.11 ± 0.02	−10.20 ± 0.30	75.57 ± 4.21	23.51 ± 5.07
FHIC-PNP**	87.3 ± 10.0	0.25 ± 0.01	−8.71 ± 0.03	78.80 ± 0.55	—
F1***	146.2 ± 2.3	0.12 ± 0.05	—	81.96 ± 3.80	4.10 ± 0.20
F2***	198.7 ± 6.0	0.16 ± 0.02	—	74.78 ± 1.90	3.74 ± 0.10
F3***	165.4 ± 3.3	0.15 ± 0.02	—	69.76 ± 2.80	3.49 ± 0.10
FST-PLGA****	187.9 ± 6.1	0.12 ± 0.01	−29.20 ± 1.60	79.30 ± 2.70	—

As reported in Table 1, the nanoformulation that we prepared has a more negative ζ-potential than that reported by Kadari et al., conferring it good colloidal stability. Moreover, we achieved a higher drug loading (DL) value, corresponding to 23.51%, that is, 6 to 8 times greater than the result reported in Sechi et al. PLGA (FIS) nanoparticles show a lower particle size than all the other herein reported, with an exception of those prepared by Kadari et al. but in every case showing a better monodispersivity, as it is evident from the lower PDI value.

To further investigate the size distribution of nanoparticles, nanotracking and AFM analysis were also performed. The nanoparticles shape is quite spherical (Supplementary Figure S2), and as it is evident from Table 2, data from the three different methods are in agreement and span in the same order of magnitude, even if the techniques are based on different physicochemical properties.

**TABLE 2:** DLS, AFM, and nanotracking analysis data of empty, PLGA (Fis), freeze-dried PLGA (Fis)*, and PLGA (Fis and Fitc) nanoparticles. The statistical analysis was performed over a population > major than 30 using SPIP statistical tool.

Nanoformulation	Z-average (NM)	Peak number (NM)	AFM diameter** (NM)	Nanotracking analysis (NM)
Empty	173.8 ± 2.0	141.60 ± 44.34	181.80 ± 28.98	136.0 ± 29.0
PLGA (Fis)	139.2 ± 4.0	105.07 ± 31.67	142.77 ± 17.29	117.2 ± 21.7
F-dried PLGA (Fis)*	152.6 ± 2.7	115.07 ± 37.38	—	—
PLGA (Fis and Fitc)	180.6 ± 2.8	139.6 ± 55.31	193.63 ± 40.71	123.6 ± 28.2

Furthermore, freeze-drying does not negatively affect the samples size after resuspension and the co-encapsulation of fisetin, and FITC does not affect the size of the nanoparticles noticeably.

**FIGURE 8:**
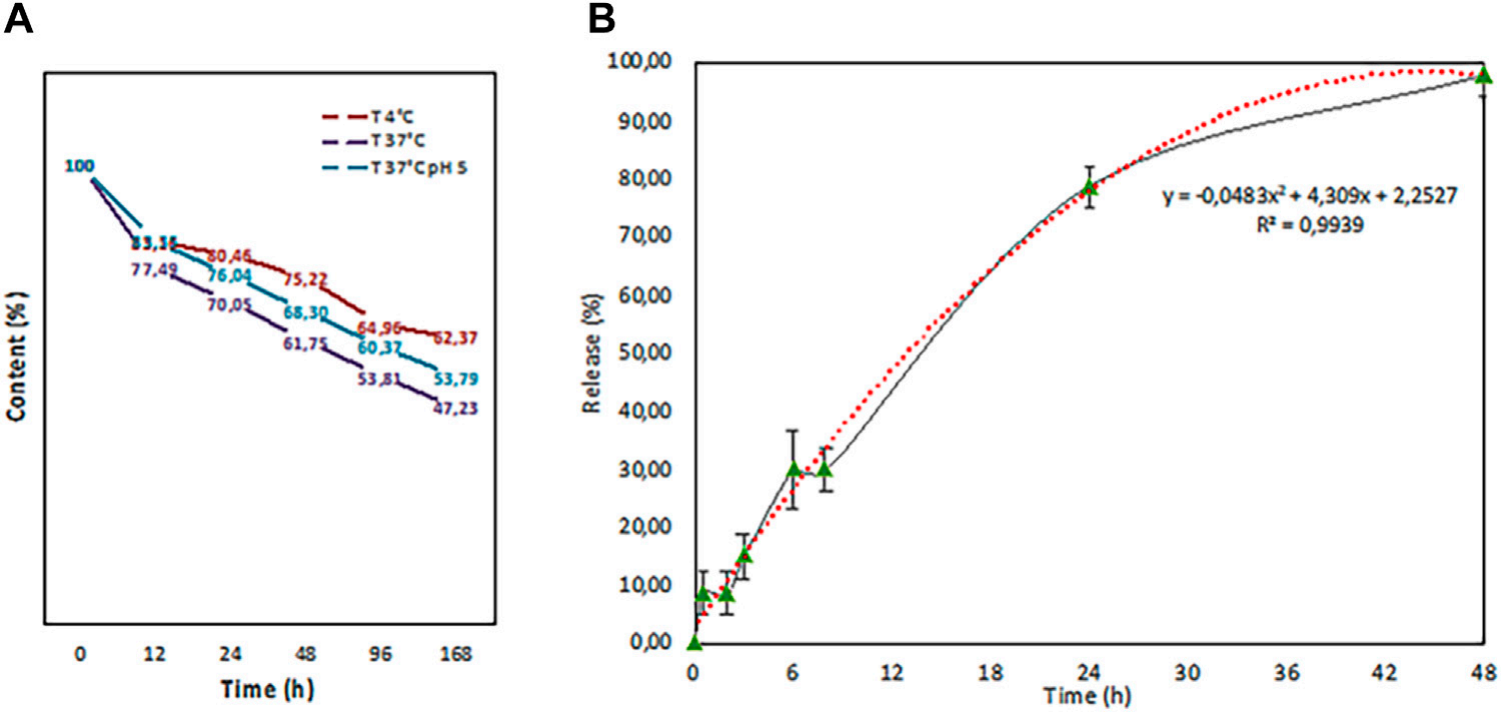
Release test performed at different temperatures (4°C (red line) and 37°C (purple line) in PBS and in citric acid pH 5 (37°C (blue line) in a final volume of 1 ml. b. In-vitro release study in 100 ml of physiological solution that fits with a second grade polynomial function curve (red dots).

We also tested the fisetin release rate of PLGA nanoparticles at different temperatures (4 and 37°C) and in citric acid pH 5 in a final 1 ml volume. As it is evident from Figure 8A, after an initial burst loss, the release kinetics dropped dramatically.

The temperature increase, as expected, leads to fast kinetics, while an acidic pH seems to slow down the loss. Then, an *in vitro* release study on the volume subsequently used for tests on intestinal epithelial tissue was carried out (Figure 8B). PLGA (Fis) NPs exerted a sustained release trend that fits with a second-degree polynomial function.

A microfluidic system containing human intestinal epithelial tissue was created to assess the ability of PLGA (Fis) to cross the intestine (Supplementary Figure S3). As expected PLGA (Fis) could cross the intestinal epithelium since the nanoparticles with negative zeta potential are taken up by Peyer’s patches and are translocated into the blood circulation (Joshi et al., 2014). Our data are shown in Table 3: after 5-h incubation, less than 5% of the initial concentration crossed the epithelium, while a value of 30% was reached within 16 h.

**TABLE 3:** Quantification of fisetin concentration after incubation with the intestinal epithelium for 5 or 16 h.

Concentration of PLGA (Fis) added to the intestinal epithelial tissue (uM)	Concentration of PLGA (Fis) crossing the epithelium after 5 h (uM)	Concentration of PLGA (Fis) crossing the epithelium after 16 h (uM)
1550 ± 2828	7344 ± 714	43617 ± 795

The fisetin amount has been calculated using a fluorescence calibration curve (Supplementary Figure S4). Moreover, PLGA (Fis) recovered after the experiment in the volume filtered by the epithelium showed a slight increase in the size, with values of approximately 190 nm. This increase in the size of PLGA (Fis) might be due to the well-known protein corona phenomenon or to some interactions occurring during the epithelial tissue crossing.

**FIGURE 9:**
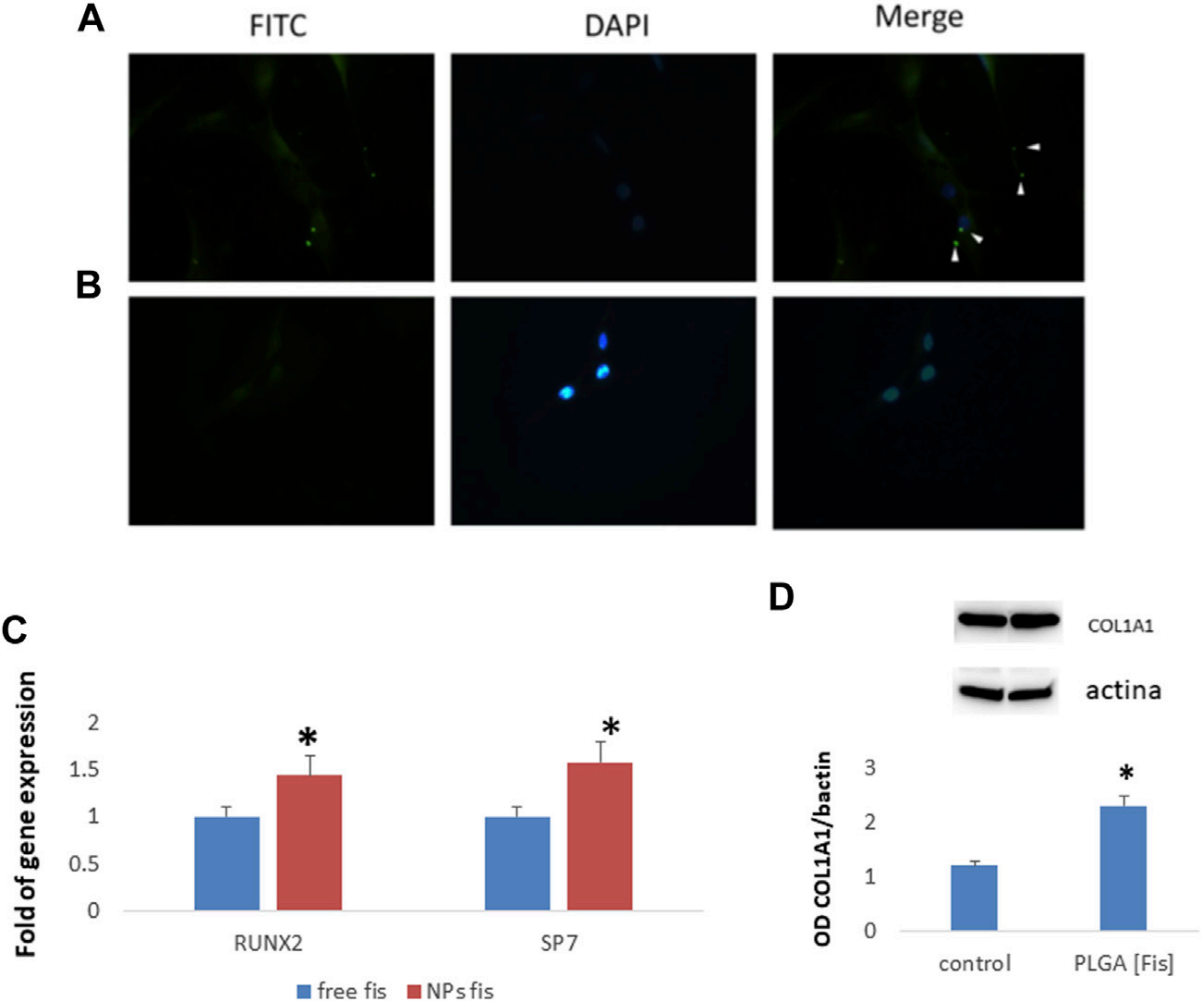
PLGA (Fis) are visible after 4 h of treatment in cultures and fisetin (in green, FITC) diffuses in intercellular spaces **(A)**. However, after 6 h, fisetin is completely absorbed, and only cellular nuclei [blue, 4′,6–diamidino-2-fenilindolo diidrocloruro (DAPI) stained nuclei] are visible **(B)**. After 7 days of osteogenic differentiation, PLGA (Fis) increased the expression of the osteogenic transcription factors RUNX2 and SP7 **(C)**. COL1A1 chain levels increased in cells treated with PLGA (Fis) compared to control **(D)**. *p < 0.05. Magnification × 40.

Subsequently, in order to test the ability of PLGA (Fis) to induce osteogenic differentiation, we cultured MSCs with FITC-PLGA (Fis). In particular, we cultured MSCs in an osteogenic medium with or without FITC-PLGA (Fis) for 7 days. Fresh FITC-PLGA (Fis)s were added to every medium change (every 2 or 3 days). Four hours after the addition of the complete medium, PLGA (Fis)s are visible in intercellular spaces (Figure 9A). After 6 h of treatment, fisetin (in green) was completely incorporated into the cells (Figure 9B).

After 7 days of osteogenic differentiation, cells were collected to perform gene expression analyses. As shown in Figure 9C, we observed that PLGA (Fis) increased the expression of the osteogenic transcription factors RUNX2 and SP7 compared to free fisetin-supplemented cells. Accordingly, COL1A1, a marker of osteogenic maturation, was higher in cells treated with PLGA (Fis) than in control (Figure 9D).

## 4 Discussion

Flavonoid intake has been associated with increased bone mineral density (Welch et al., 2012; Léotoing et al., 2014). *In vitro* models have demonstrated the ability of flavonoids to modulate bone formation by acting on osteoblasts (bone-forming cells) (Trzeciakiewicz et al., 2009). It is well known that osteoblasts arise from mesenchymal stem cells through a differentiation process and that various factors/conditions may affect this process (Valenti M. T. et al., 2018; Valenti et al., 2018 MT.). Extracellular signaling such as Wnt, PTH, and hedgehog acts by modulating the expression of the osteogenic master gene RUNX2 (Soltanoff et al., 2009). RUNX2 plays a central role in osteogenesis; Runx2 mutations cause cleidocranial dysplasia, a skeletal disorder (Lee et al., 1997). The polyphenol fisetin has been shown to influence a wide variety of cellular processes *in vitro*. In fact, fisetin exerts a proapoptotic activity on cancer cells (Sung et al., 2007; Khan et al., 2008a); it acts as an antioxidant in neuronal cells (Ishige et al., 2001; Hanneken et al., 2006), as well as an anti-inflammatory agent in macrophages and fibroblasts (Liu et al., 2010; Funakoshi-Tago et al., 2011). *In vivo*, it has been shown that fisetin counteracts rheumatoid arthritis (Lee et al., 2009) and inflammation-induced bone loss (Léotoing et al., 2013). In addition, it has been reported that fisetin promotes osteoblast differentiation through GSK-3β phosphorylation and β-catenin activation (20). Therefore, these previous works suggest its potential to counteract skeletal diseases.

Our data confirmed that fisetin is able to promote the osteogenic maturation of MSCs. Several studies have pursued the identification of substances capable of promoting osteogenesis and improving bone quality for the prevention/reduction of skeletal pathologies. Among these molecules, ascorbic acid (AsA) regulates collagen deposition and promotes the differentiation of MSCs to osteoblasts and chondrocytes (D’Aniello et al., 2017), and the pigment epithelium-derived factor (PEDF) inhibits adipogenesis and promotes osteogenesis of human MSCs (Gattu et al., 2013). In our study, we observed that fisetin as well induces the expression of osteogenic genes at comparable levels. In some cases, for example, for COL1A1 and SPARC expression at 14 days of differentiation, we observed higher gene expression levels in fisetin-treated MSCs.

Data obtained by using zebrafish as the *in vivo* model confirm that fisetin promotes bone formation by modulating bone-specific genes. As reported, in zebrafish, most bones show the sequential gene expression required for osteogenic differentiation in mammals (Li et al., 2009). As in mammals, three stages can be recognized in the osteogenic differentiation: an early stage characterized by runx2a and runx2b expression; an intermediate stage characterized by osterix gene expression; and a maturation stage characterized by the expression of osteocalcin, osteonectin, collagens, and other bone matrix genes (Li et al., 2009; Knopf et al., 2011). Zebrafish has been proposed as a useful *in vivo* model for studying cell signaling in bone (Valenti et al., 2020a) and for evaluating the effects of bone treatments (Valenti et al., 2020b). Zebrafish are frequently used in developmental biology (Suniaga et al., 2018). However, the effects of osteoanabolic supplementation on mature zebrafish skeleton have been poorly investigated. Experiments focusing on skeletal changes following stimulation in adult zebrafish could allow the evaluation of molecular treatments in aging-induced bone loss. In our study, we found that fisetin promotes mineralization in aged models by investigating osteogenic gene expression modulation and mineralization in 15- to 20-month-old zebrafish.

It is well known that different skeletal diseases or conditions show deregulated bone formation, for example, osteoporosis (Carbonare et al., 2009; Valenti et al., 2011; Benisch et al., 2012; Föger-Samwald et al., 2014; Cheng et al., 2019, 2), and systemic diseases like diabetes (Lu et al., 2003), aging-associated bone loss (De Martinis et al., 2006; Li et al., 2015), drug-induced bone disorders (Henneicke et al., 2014), osteogenesis imperfecta (Kaneto et al., 2017; Infante et al., 2021), and cleidocranial dysplasia (Dalle Carbonare et al., 2021).

In particular, we reported that in patients affected by CCD, an autosomal dominant inherited skeletal disease, RUNX2 mutations impair osteogenic differentiation (Dalle Carbonare et al., 2021). In the current study, we found that fisetin enhances osteoblast maturation in samples of pediatric patients affected by cleidocranial dysplasia. In fact, we observed that fisetin stimulates the expression of SPARC, a downstream osteogenic gene associated with the maturation phase of osteogenic differentiation. However, it would be interesting to evaluate the effect of fisetin’s in RUNX2 KO animal models in order to understand fisetin’s role and action in CCD pathology.

Léotoing et al. observed that fisetin increased Runx2 transcriptional activity (Léotoing et al., 2014). In addition, we observed that ROS levels in MSCs during osteogenic differentiation in the presence of fisetin were reduced during the late phase of differentiation, while they were not affected during the first phase. It has been demonstrated that mineralization matches with a reduction of intracellular ROS levels (Atashi et al., 2015; Mohamad et al., 2021) and that oxidative stress causes impairment in osteogenic differentiation of MSCs (Hussein and Sina, 2020). Therefore, by considering the antioxidant properties of fisetin (Khan et al., 2013), we may consider this advantage for osteogenic maturation. We did not observe reduction in ROS levels during the early phase of differentiation probably as ROS accumulation occurs during differentiation (Valle-Prieto and Conget, 2010; Sart et al., 2015).

All these findings clearly show that fisetin can be considered an excellent molecule for bone formation promotion, suggesting its use for relief in bone diseases caused by osteogenic differentiation deregulation. Fisetin’s drawbacks however are poor water solubility and chemical instability (Sechi et al., 2016). Indeed, fisetin has been successfully encapsulated into PLGA in previous works by Sechi et al. and Kadari et al., using nanoprecipitation and double emulsion methods. Alternatively, in the present article, we report the use of the single emulsion displacement method, obtaining similar results in terms of size and encapsulation efficiency (EE), as reported in Table 1. In addition, the nanoformulation that we performed has a more negative ζ-potential than that reported by Kadari et al., conferring it good colloidal stability. Moreover, we succeeded in achieving a higher drug loading (DL) value, corresponding to 23.51%, from 6 to 8 times greater than the result reported by Sechi et al.

In particular, in the first reported study, a double emulsion was made, specifically fisetin was complexed with hydroxyl propyl beta-cyclodextrin to increase its solubility in water, and then it was emulsified to obtain the PLGA nanoparticles. In the second reported study, the authors used a nanoprecipitation and two different polymers (PLGA-peg and polycapryl lactone). However, in our study, we used a single emulsion; compared to those previously reported, the method we applied is simpler. Notably, fisetin is not complexed with other molecules, and it interacts directly with PLGA (so “pure” fisetin is released, instead of a fisetin-excipient complex). Remarkably, the effects of nanoencapsulated fisetin on osteogenic differentiation and the percentage of intestinal filtration of PLGA-encapsulated fisetin have not been explored previously. In our study, we observed increased osteogenic differentiation in MSCs supplemented with PLGA-encapsulated fisetin compared to fisetin alone, at equal fisetin concentrations. We believe that this last finding is important considering that fisetin is easily degradable if not protected within nanoparticles.

## 5 Conclusion

Our findings consolidate the knowledge about fisetin properties and demonstrate that fisetin induces osteogenic gene expression at similar or higher levels, if compared to other pro-osteogenic molecules such as AsA and PEDF.

We also observed that fisetin promotes mineralization in mature zebrafish, suggesting that fisetin might be useful in reducing aging-induced bone loss. In addition, the increased expression of the osteogenic maturation SPARC gene observed in fisetin-treated CCD “*ex vivo*” cells prompts the employment of this flavonoid for counteracting bone diseases associated with osteogenesis dysregulation. Importantly, we generated PLGA (Fis) with elevated drug loading and were able to cross the human intestinal epithelial tissue.

## Data Availability

The original contributions presented in the study are included in the article/Supplementary Materials, further inquiries can be directed to the corresponding author.

## References

[B1] Arai Y. Watanabe S. Kimira M. Shimoi K. Mochizuki R. Kinae N. (2000). Dietary Intakes of Flavonols, Flavones and Isoflavones by Japanese Women and the Inverse Correlation between Quercetin Intake and Plasma LDL Cholesterol Concentration. J. Nutr. 130, 2243–2250. 10.1093/jn/130.9.2243 10958819

[B2] Ardeshirylajimi A. Soleimani M. Hosseinkhani S. Parivar K. Yaghmaei P. (2014). A Comparative Study of Osteogenic Differentiation Human Induced Pluripotent Stem Cells and Adipose Tissue Derived Mesenchymal Stem Cells. Cell J 16, 235–244. 24611148 PMC4204190

[B3] Atashi F. Modarressi A. Pepper M. S. (2015). The Role of Reactive Oxygen Species in Mesenchymal Stem Cell Adipogenic and Osteogenic Differentiation: A Review. Stem Cell Dev 24, 1150–1163. 10.1089/scd.2014.0484 PMC4424969 25603196

[B4] Benisch P. Schilling T. Klein-Hitpass L. Frey S. P. Seefried L. Raaijmakers N. (2012). The Transcriptional Profile of Mesenchymal Stem Cell Populations in Primary Osteoporosis Is Distinct and Shows Overexpression of Osteogenic Inhibitors. PLoS ONE 7, e45142. 10.1371/journal.pone.0045142 23028809 PMC3454401

[B5] Brugnara C. De Franceschi L. (1993). Effect of Cell Age and Phenylhydrazine on the Cation Transport Properties of Rabbit Erythrocytes. J. Cel. Physiol. 154, 271–280. 10.1002/jcp.1041540209 8381125

[B6] Cecconi D. Brandi J. Manfredi M. Serena M. Dalle Carbonare L. Deiana M. (2019). Runx2 Stimulates Neoangiogenesis through the Runt Domain in Melanoma. Sci. Rep. 9, 8052. 10.1038/s41598-019-44552-1 31142788 PMC6541657

[B7] Cederroth C. R. Nef S. (2009). Soy, Phytoestrogens and Metabolism: A Review. Mol. Cel Endocrinol 304, 30–42. 10.1016/j.mce.2009.02.027 19433245

[B8] Cheng F. Yang M. M. Yang R. H. (2019). MiRNA-365a-3p Promotes the Progression of Osteoporosis by Inhibiting Osteogenic Differentiation via Targeting RUNX2. Eur. Rev. Med. Pharmacol. Sci. 23, 7766–7774. 10.26355/eurrev_201909_18986 31599402

[B9] D'Aniello C. Cermola F. Patriarca E. J. Minchiotti G. (2017). Vitamin C in Stem Cell Biology: Impact on Extracellular Matrix Homeostasis and Epigenetics. Stem Cell Int 2017, 8936156–8936216. 10.1155/2017/8936156 PMC5415867 28512473

[B10] Dalle Carbonare L. Antoniazzi F. Gandini A. Orsi S. Bertacco J. Li Vigni V. (2021). Two Novel C-Terminus RUNX2 Mutations in Two Cleidocranial Dysplasia (CCD) Patients Impairing P53 Expression. Int. J. Mol. Sci. 22, 10336. 10.3390/ijms221910336 34638677 PMC8508986

[B11] Dalle Carbonare L. Mottes M. Cheri S. Deiana M. Zamboni F. Gabbiani D. (2019). Increased Gene Expression of RUNX2 and SOX9 in Mesenchymal Circulating Progenitors Is Associated with Autophagy during Physical Activity. Oxid Med. Cel Longev 2019, 3356–3365. 10.1155/2019/8426259 PMC6815530 31737174

[B12] Dalle Carbonare L. Valenti M. T. Zanatta M. Donatelli L. Lo Cascio V. (2009). Circulating Mesenchymal Stem Cells with Abnormal Osteogenic Differentiation in Patients with Osteoporosis. Arthritis Rheum. 60, 3356–3365. 10.1002/art.24884 19877060

[B13] De Franceschi L. Iolascon A. Taher A. Cappellini M. D. (2017). Clinical Management of Iron Deficiency Anemia in Adults: Systemic Review on Advances in Diagnosis and Treatment. Eur. J. Intern. Med. 42, 16–23. 10.1016/j.ejim.2017.04.018 28528999

[B14] de Franceschi L. Turrini F. Honczarenko M. Ayi K. Rivera A. Fleming M. D. (2004). *In Vivo* reduction of Erythrocyte Oxidant Stress in a Murine Model of Beta-Thalassemia. Haematologica 89, 1287–1298. 15531450

[B15] De Martinis M. Di Benedetto M. C. Mengoli L. P. Ginaldi L. (2006). Senile Osteoporosis: Is it an Immune-Mediated Disease? Inflamm. Res. 55, 399–404. 10.1007/s00011-006-6034-x 17109066

[B16] Deiana M. Malerba G. Dalle Carbonare L. Cheri S. Patuzzo C. Tsenov G. (2019). Physical Activity Prevents Cartilage Degradation: A Metabolomics Study Pinpoints the Involvement of Vitamin B6. Cells 8, 1374. 10.3390/cells8111374 PMC6912200 31683926

[B17] Du S. J. Frenkel V. Kindschi G. Zohar Y. (2001). Visualizing Normal and Defective Bone Development in Zebrafish Embryos Using the Fluorescent Chromophore Calcein. Dev. Biol. 238, 239–246. 10.1006/dbio.2001.0390 11784007

[B18] Ducy P. Zhang R. Geoffroy V. Ridall A. L. Karsenty G. (1997). Osf2/Cbfa1: A Transcriptional Activator of Osteoblast Differentiation. Cell 89, 747–754. 10.1016/S0092-8674(00)80257-3 9182762

[B19] Föger-Samwald U. Patsch J. M. Schamall D. Alaghebandan A. Deutschmann J. Salem S. (2014). Molecular Evidence of Osteoblast Dysfunction in Elderly Men with Osteoporotic Hip Fractures. Exp. Gerontol. 57, 114–121. 10.1016/j.exger.2014.05.014 24862290

[B20] Friis Jan J. (2009). Scanning Probe Image Processor 4.8: User’s and Reference Guide. Lyngby, Denmark: CreateSpace Independent Publishing Platform.

[B21] Funakoshi-Tago M. Nakamura K. Tago K. Mashino T. Kasahara T. (2011). Anti-inflammatory Activity of Structurally Related Flavonoids, Apigenin, Luteolin and Fisetin. Int. Immunopharmacol 11, 1150–1159. 10.1016/j.intimp.2011.03.012 21443976

[B22] Gaglio S. C. De Rosa C. Piccinelli F. Romeo A. Perduca M. (2019). Complexes of Rare Earth Ions Embedded in Poly(lactic-Co-Glycolic Acid) (PLGA) Nanoparticles: Characterization and Spectroscopic Study. Opt. Mater. 94, 249–256. 10.1016/j.optmat.2019.05.034

[B23] Gattu A. K. Swenson E. S. Iwakiri Y. Samuel V. T. Troiano N. Berry R. (2013). Determination of Mesenchymal Stem Cell Fate by Pigment Epithelium-Derived Factor (PEDF) Results in Increased Adiposity and Reduced Bone mineral Content. FASEB j 27, 4384–4394. 10.1096/fj.13-232900 23887690 PMC3804749

[B24] Hanneken A. Lin F. F. Johnson J. Maher P. (2006). Flavonoids Protect Human Retinal Pigment Epithelial Cells from Oxidative-Stress-Induced Death. Invest. Ophthalmol. Vis. Sci. 47, 3164–3177. 10.1167/iovs.04-1369 16799064

[B25] Henneicke H. Gasparini S. J. Brennan-Speranza T. C. Zhou H. Seibel M. J. (2014). Glucocorticoids and Bone: Local Effects and Systemic Implications. Trends Endocrinol. Metab. 25, 197–211. 10.1016/j.tem.2013.12.006 24418120

[B26] Hussein A. M. Sina M. (2020). p-Nonylphenol Impairment of Osteogenic Differentiation of Mesenchymal Stem Cells Was Found to Be Due to Oxidative Stress and Down-Regulation of RUNX2 and BMP. Endocr. Metab. Immune Disord. Drug Targets 20, 1336–1346. 10.2174/1871530320666200505114058 32368982

[B27] Infante A. Gener B. Vázquez M. Olivares N. Arrieta A. Grau G. (2021). Reiterative Infusions of MSCs Improve Pediatric Osteogenesis Imperfecta Eliciting a Pro-osteogenic Paracrine Response: TERCELOI Clinical Trial. Clin. Transl Med. 11, e265. 10.1002/ctm2.265 33463067 PMC7805402

[B28] Ishige K. Schubert D. Sagara Y. (2001). Flavonoids Protect Neuronal Cells from Oxidative Stress by Three Distinct Mechanisms. Free Radic. Biol. Med. 30, 433–446. 10.1016/S0891-5849(00)00498-6 11182299

[B29] Joshi G. Kumar A. Sawant K. (2014). Enhanced Bioavailability and Intestinal Uptake of Gemcitabine HCl Loaded PLGA Nanoparticles after Oral Delivery. Eur. J. Pharm. Sci. 60, 80–89. 10.1016/j.ejps.2014.04.014 24810394

[B30] Kadari A. Gudem S. Kulhari H. Bhandi M. M. Borkar R. M. Kolapalli V. R. (2017). Enhanced Oral Bioavailability and Anticancer Efficacy of Fisetin by Encapsulating as Inclusion Complex with HPβCD in Polymeric Nanoparticles. Drug Deliv. 24, 224–232. 10.1080/10717544.2016.1245366 28156161 PMC8241160

[B31] Kaneto C. M. Pereira Lima P. S. Prata K. L. dos Santos J. L. de Pina Neto J. M. Panepucci R. A. (2017). Gene Expression Profiling of Bone Marrow Mesenchymal Stem Cells from Osteogenesis Imperfecta Patients during Osteoblast Differentiation. Eur. J. Med. Genet. 60, 326–334. 10.1016/j.ejmg.2017.04.003 28396251

[B32] Khan N. Afaq F. Syed D. N. Mukhtar H. (2008a). Fisetin, a Novel Dietary Flavonoid, Causes Apoptosis and Cell Cycle Arrest in Human Prostate Cancer LNCaP Cells. Carcinogenesis 29, 1049–1056. 10.1093/carcin/bgn078 18359761 PMC2902387

[B33] Khan N. Asim M. Afaq F. Abu Zaid M. Mukhtar H. (2008b). A Novel Dietary Flavonoid Fisetin Inhibits Androgen Receptor Signaling and Tumor Growth in Athymic Nude Mice. Cancer Res. 68, 8555–8563. 10.1158/0008-5472.CAN-08-0240 18922931 PMC2954499

[B34] Khan N. Syed D. N. Ahmad N. Mukhtar H. (2013). Fisetin: A Dietary Antioxidant for Health Promotion. Antioxid. Redox Signal. 19, 151–162. 10.1089/ars.2012.4901 23121441 PMC3689181

[B35] Kimmel C. B. Ballard W. W. Kimmel S. R. Ullmann B. Schilling T. F. (1995). Stages of Embryonic Development of the Zebrafish. Dev. Dyn. 203, 253–310. 10.1002/aja.1002030302 8589427

[B36] Knopf F. Hammond C. Chekuru A. Kurth T. Hans S. Weber C. W. (2011). Bone Regenerates via Dedifferentiation of Osteoblasts in the Zebrafish Fin. Dev. Cel 20, 713–724. 10.1016/j.devcel.2011.04.014 21571227

[B37] Komori T. (2010). Regulation of Bone Development and Extracellular Matrix Protein Genes by RUNX2. Cell Tissue Res 339, 189–195. 10.1007/s00441-009-0832-8 19649655

[B38] Kular J. Tickner J. Chim S. M. Xu J. (2012). An Overview of the Regulation of Bone Remodelling at the Cellular Level. Clin. Biochem. 45, 863–873. 10.1016/j.clinbiochem.2012.03.021 22465238

[B39] Lee B. Thirunavukkarasu K. Zhou L. Pastore L. Baldini A. Hecht J. (1997). Missense Mutations Abolishing DNA Binding of the Osteoblast-specific Transcription Factor OSF2/CBFA1 in Cleidocranial Dysplasia. Nat. Genet. 16, 307–310. 10.1038/ng0797-307 9207800

[B40] Lee J. D. Huh J. E. Jeon G. Yang H. R. Woo H. S. Choi D. Y. (2009). Flavonol-rich RVHxR from Rhus Verniciflua Stokes and its Major Compound Fisetin Inhibits Inflammation-Related Cytokines and Angiogenic Factor in Rheumatoid Arthritic Fibroblast-like Synovial Cells and *In Vivo* Models. Int. Immunopharmacol 9, 268–276. 10.1016/j.intimp.2008.11.005 19111632

[B41] Léotoing L. Davicco M. J. Lebecque P. Wittrant Y. Coxam V. (2014). The Flavonoid Fisetin Promotes Osteoblasts Differentiation through Runx2 Transcriptional Activity. Mol. Nutr. Food Res. 58, 1239–1248. 10.1002/mnfr.201300836 24535991

[B42] Léotoing L. Wauquier F. Guicheux J. Miot-Noirault E. Wittrant Y. Coxam V. (2013). The Polyphenol Fisetin Protects Bone by Repressing NF-Κb and MKP-1-dependent Signaling Pathways in Osteoclasts. PLoS ONE 8, e68388. 10.1371/journal.pone.0068388 23861901 PMC3701685

[B43] Li C. J. Cheng P. Liang M. K. Chen Y. S. Lu Q. Wang J. Y. (2015). MicroRNA-188 Regulates Age-Related Switch between Osteoblast and Adipocyte Differentiation. J. Clin. Invest. 125, 1509–1522. 10.1172/JCI77716 25751060 PMC4396470

[B44] Li N. Felber K. Elks P. Croucher P. Roehl H. H. (2009). Tracking Gene Expression during Zebrafish Osteoblast Differentiation. Dev. Dyn. 238, 459–466. 10.1002/dvdy.21838 19161246

[B45] Liu S. H. Lin C. H. Hung S. K. Chou J. H. Chi C. W. Fu S. L. (2010). Fisetin Inhibits Lipopolysaccharide-Induced Macrophage Activation and Dendritic Cell Maturation. J. Agric. Food Chem. 58, 10831–10839. 10.1021/jf1017093 20923145

[B46] Liu W. Y. Lin C. C. Hsieh Y. S. Wu Y. T. (2021). Nanoformulation Development to Improve the Biopharmaceutical Properties of Fisetin Using Design of Experiment Approach. Molecules 26, 3031. 10.3390/molecules26103031 34069585 PMC8160650

[B47] Lu H. Kraut D. Gerstenfeld L. C. Graves D. T. (2003). Diabetes Interferes with the Bone Formation by Affecting the Expression of Transcription Factors that Regulate Osteoblast Differentiation. Endocrinology 144, 346–352. 10.1210/en.2002-220072 12488363

[B48] Makadia H. K. Siegel S. J. (2011). Poly Lactic-Co-Glycolic Acid (PLGA) as Biodegradable Controlled Drug Delivery Carrier. Polymers (Basel) 3, 1377–1397. 10.3390/polym3031377 22577513 PMC3347861

[B49] Marie P. J. (2008). Transcription Factors Controlling Osteoblastogenesis. Arch. Biochem. Biophys. 473, 98–105. 10.1016/j.abb.2008.02.030 18331818

[B50] Mohamad S. A. Milward M. R. Hadis M. A. Kuehne S. A. Cooper P. R. (2021). Photobiomodulation of Mineralisation in Mesenchymal Stem Cells. Photochem. Photobiol. Sci. 20, 699–714. 10.1007/s43630-021-00047-5 33945145

[B51] Molagoda I. M. N. Kang C. H. Lee M. H. Choi Y. H. Lee C. M. Lee S. (2021). Fisetin Promotes Osteoblast Differentiation and Osteogenesis through GSK-3β Phosphorylation at Ser9 and Consequent β-catenin Activation, Inhibiting Osteoporosis. Biochem. Pharmacol. 192, 114676. 10.1016/j.bcp.2021.114676 34256044

[B52] Sakata-Haga H. Uchishiba M. Shimada H. Tsukada T. Mitani M. Arikawa T. (2018). A Rapid and Nondestructive Protocol for Whole-Mount Bone Staining of Small Fish and Xenopus. Sci. Rep. 8, 7453. 10.1038/s41598-018-25836-4 29748567 PMC5945591

[B53] Sart S. Song L. Li Y. (2015). Controlling Redox Status for Stem Cell Survival, Expansion, and Differentiation. Oxid Med. Cel Longev 2015, 105135. 10.1155/2015/105135 PMC4530287 26273419

[B54] Sechi M. Syed D. N. Pala N. Mariani A. Marceddu S. Brunetti A. (2016). Nanoencapsulation of Dietary Flavonoid Fisetin: Formulation and *In Vitro* Antioxidant and α-glucosidase Inhibition Activities. Mater. Sci. Eng. C Mater. Biol. Appl. 68, 594–602. 10.1016/j.msec.2016.06.042 27524059

[B55] Soltanoff C. S. Yang S. Chen W. Li Y. P. (2009). Signaling Networks that Control the Lineage Commitment and Differentiation of Bone Cells. Crit. Rev. Eukaryot. Gene Expr. 19, 1–46. 10.1615/CritRevEukarGeneExpr.v19.i1.10 19191755 PMC3392028

[B56] Sung B. Pandey M. K. Aggarwal B. B. (2007). Fisetin, an Inhibitor of Cyclin-dependent Kinase 6, Down-Regulates Nuclear Factor-Κb-Regulated Cell Proliferation, Antiapoptotic and Metastatic Gene Products through the Suppression of TAK-1 and Receptor-Interacting Protein-Regulated IκBα Kinase Activation. Mol. Pharmacol. 71, 1703–1714. 10.1124/mol.107.034512 17387141

[B57] Suniaga S. Rolvien T. vom Scheidt A. Fiedler I. A. K. Bale H. A. Huysseune A. (2018). Increased Mechanical Loading through Controlled Swimming Exercise Induces Bone Formation and Mineralization in Adult Zebrafish. Sci. Rep. 8, 3646. 10.1038/s41598-018-21776-1 29483529 PMC5826918

[B58] Trzeciakiewicz A. Habauzit V. Horcajada M. N. (2009). When Nutrition Interacts with Osteoblast Function: Molecular Mechanisms of Polyphenols. Nutr. Res. Rev. 22, 68–81. 10.1017/S095442240926402X 19243669

[B59] Valenti M. T. Garbin U. Pasini A. Zanatta M. Stranieri C. Manfro S. (2011). Role of Ox-PAPCs in the Differentiation of Mesenchymal Stem Cells (MSCs) and Runx2 and PPARγ2 Expression in MSCs-like of Osteoporotic Patients. PLoS ONE 6, e20363. 10.1371/journal.pone.0020363 21674037 PMC3108593

[B60] Valenti M. T. Marchetto G. Mottes M. Dalle Carbonare L. (2020a). Zebrafish: A Suitable Tool for the Study of Cell Signaling in Bone. Cells 9, 1911. 10.3390/cells9081911 PMC7465296 32824602

[B61] Valenti M. T. Marchetto G. Perduca M. Tiso N. Mottes M. Dalle Carbonare L. (2020b). BEL β-Trefoil Reduces the Migration Ability of RUNX2 Expressing Melanoma Cells in Xenotransplanted Zebrafish. Molecules 25, 1270. 10.3390/molecules25061270 PMC7143993 32168858

[B62] Valenti M. T. Mottes M. Cheri S. Deiana M. Micheletti V. Cosaro E. (2018b). Runx2 Overexpression Compromises Bone Quality in Acromegalic Patients. Endocr. Relat. Cancer 25, 269–277. 10.1530/ERC-17-0523 29295822

[B63] Valenti M. T. Dalle Carbonare L. Mottes M. (2018a). Role of microRNAs in Progenitor Cell Commitment and Osteogenic Differentiation in Health and Disease (Review). Int. J. Mol. Med. 41, 2441–2449. 10.3892/ijmm.2018.3452 29393379

[B64] Valle-Prieto A. Conget P. A. (2010). Human Mesenchymal Stem Cells Efficiently Manage Oxidative Stress. Stem Cell Dev 19, 1885–1893. 10.1089/scd.2010.0093 20380515

[B65] Venturi G. Gandini A. Monti E. Dalle Carbonare L. Corradi M. Vincenzi M. (2012). Lack of Expression of SERPINF1, the Gene Coding for Pigment Epithelium-Derived Factor, Causes Progressively Deforming Osteogenesis Imperfecta with normal Type I Collagen. J. Bone Miner Res. 27, 723–728. 10.1002/jbmr.1480 22113968

[B66] Welch A. MacGregor A. Jennings A. Fairweather-Tait S. Spector T. Cassidy A. (2012). Habitual Flavonoid Intakes Are Positively Associated with Bone mineral Density in Women. J. Bone Miner Res. 27, 1872–1878. 10.1002/jbmr.1649 22549983

[B67] Whitlock K. E. Westerfield M. (2000). The Olfactory Placodes of the Zebrafish Form by Convergence of Cellular fields at the Edge of the Neural Plate. Development 127, 3645–3653. 10.1242/dev.127.17.3645 10934010

[B68] Xu L. He X. Zhou Y. Yu K. Yuan M. Zhang Q. (2021). Connectivity Map Analysis Identifies Fisetin as a Treatment Compound for Osteoporosis through Activating the PI3K-AKT Signaling Pathway in Mouse Pre-osteoblastic MC3T3-E1 Cells. CPB 22, 2038–2047. 10.2174/1389201022666210301141238 33645479

[B69] Zheng W. Feng Z. You S. Zhang H. Tao Z. Wang Q. (2017). Fisetin Inhibits IL-1β-induced Inflammatory Response in Human Osteoarthritis Chondrocytes through Activating SIRT1 and Attenuates the Progression of Osteoarthritis in Mice. Int. Immunopharmacol 45, 135–147. 10.1016/j.intimp.2017.02.009 28213268

