# Supplementary material for: Fisetin: An Integrated Approach to Identify a Strategy Promoting Osteogenesis
Source: Front Pharmacol. 2022 May 16;13:890693. doi: 10.3389/fphar.2022.890693 (PMC9149166; doi:10.3389/fphar.2022.890693)
Supplement: Supplementary file 1 [file Presentation1.PPTX]

## Slide 1
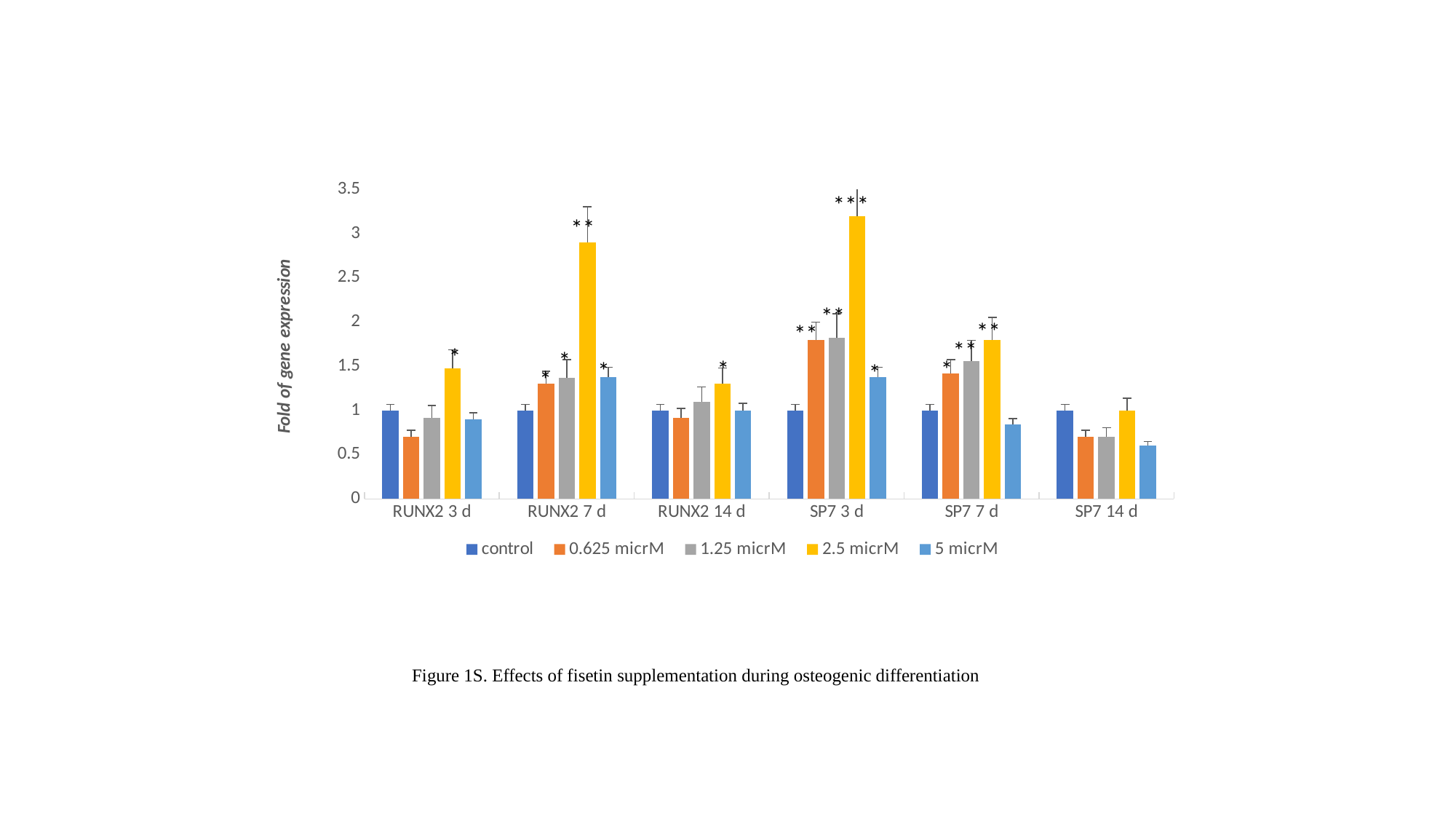

### Chart
| Category | control | 0.625 micrM | 1.25 micrM | 2.5 micrM | 5 micrM |
|---|---|---|---|---|---|
| RUNX2 3 d | 1.0 | 0.7 | 0.92 | 1.48 | 0.9 |
| RUNX2 7 d | 1.0 | 1.3 | 1.37 | 2.9 | 1.38 |
| RUNX2 14 d | 1.0 | 0.92 | 1.1 | 1.3 | 1.0 |
| SP7 3 d | 1.0 | 1.8 | 1.82 | 3.2 | 1.38 |
| SP7 7 d | 1.0 | 1.42 | 1.56 | 1.8 | 0.84 |
| SP7 14 d | 1.0 | 0.7 | 0.7 | 1.0 | 0.6 |***
**
**
**
**
**
*
*
*
*
*
*
*
Figure 1S. Effects of fisetin supplementation during osteogenic differentiation

## Slide 2
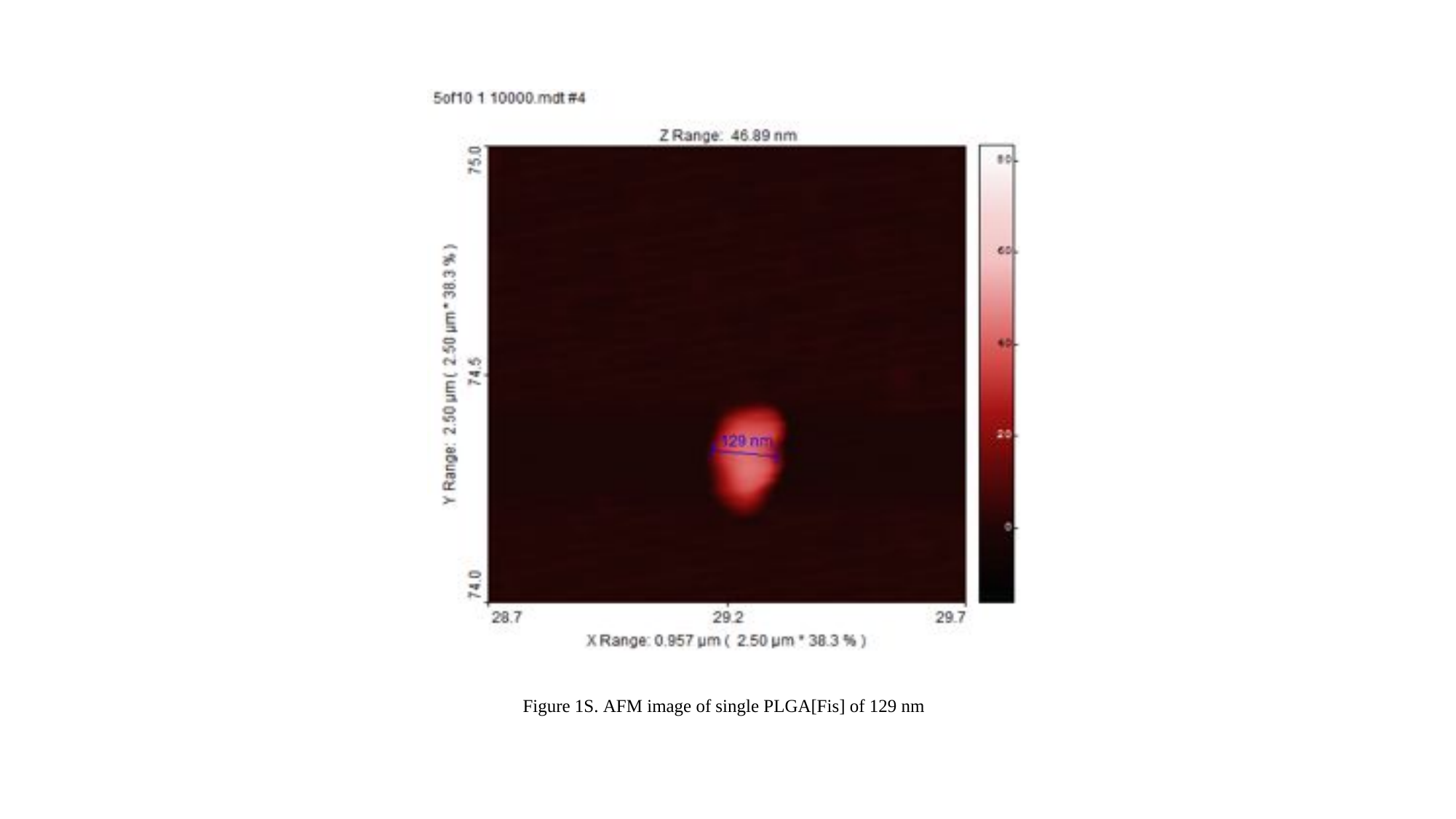

Figure 1S. AFM image of single PLGA[Fis] of 129 nm

## Slide 3
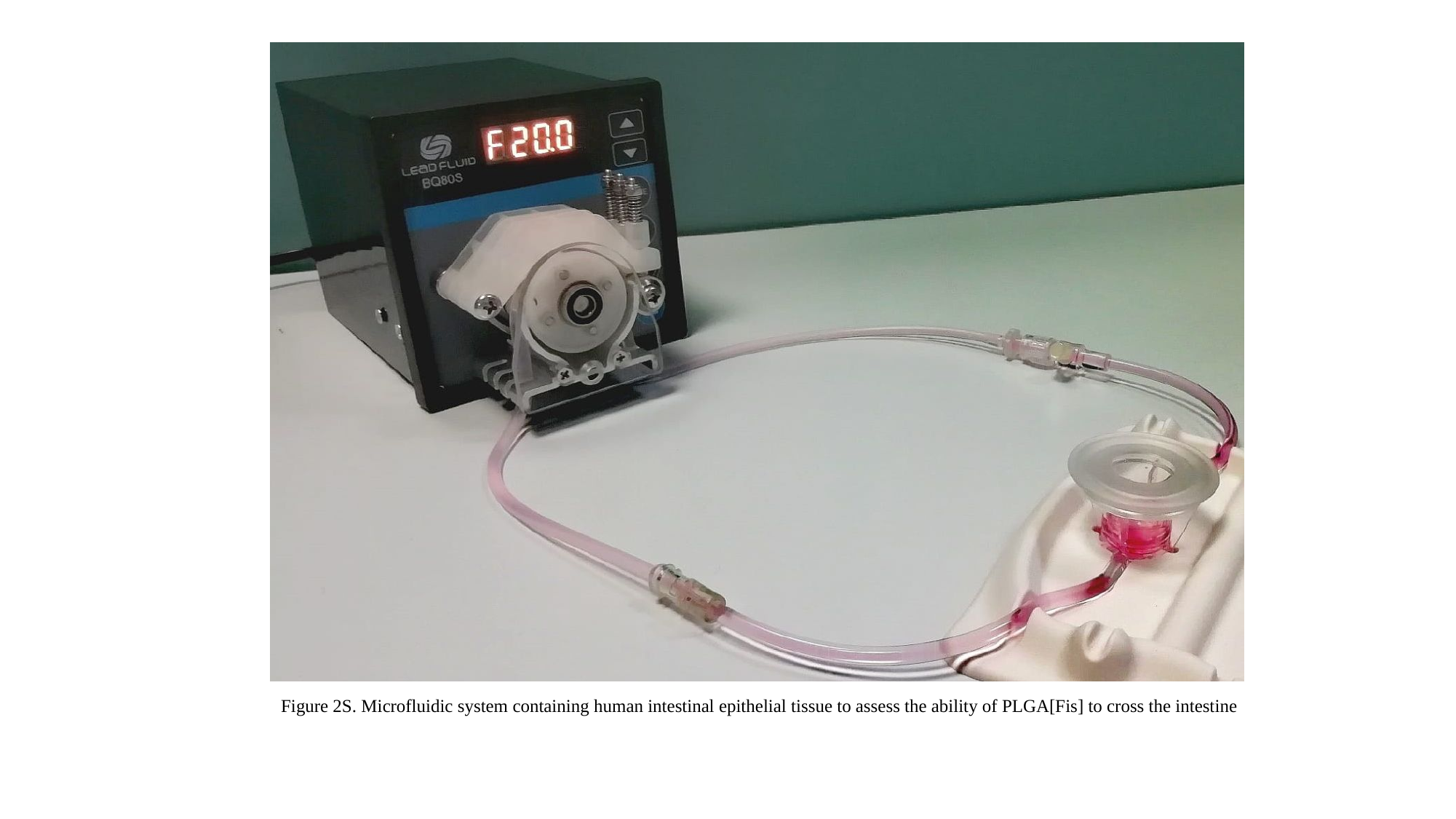

Figure 2S. Microfluidic system containing human intestinal epithelial tissue to assess the ability of PLGA[Fis] to cross the intestine

## Slide 4
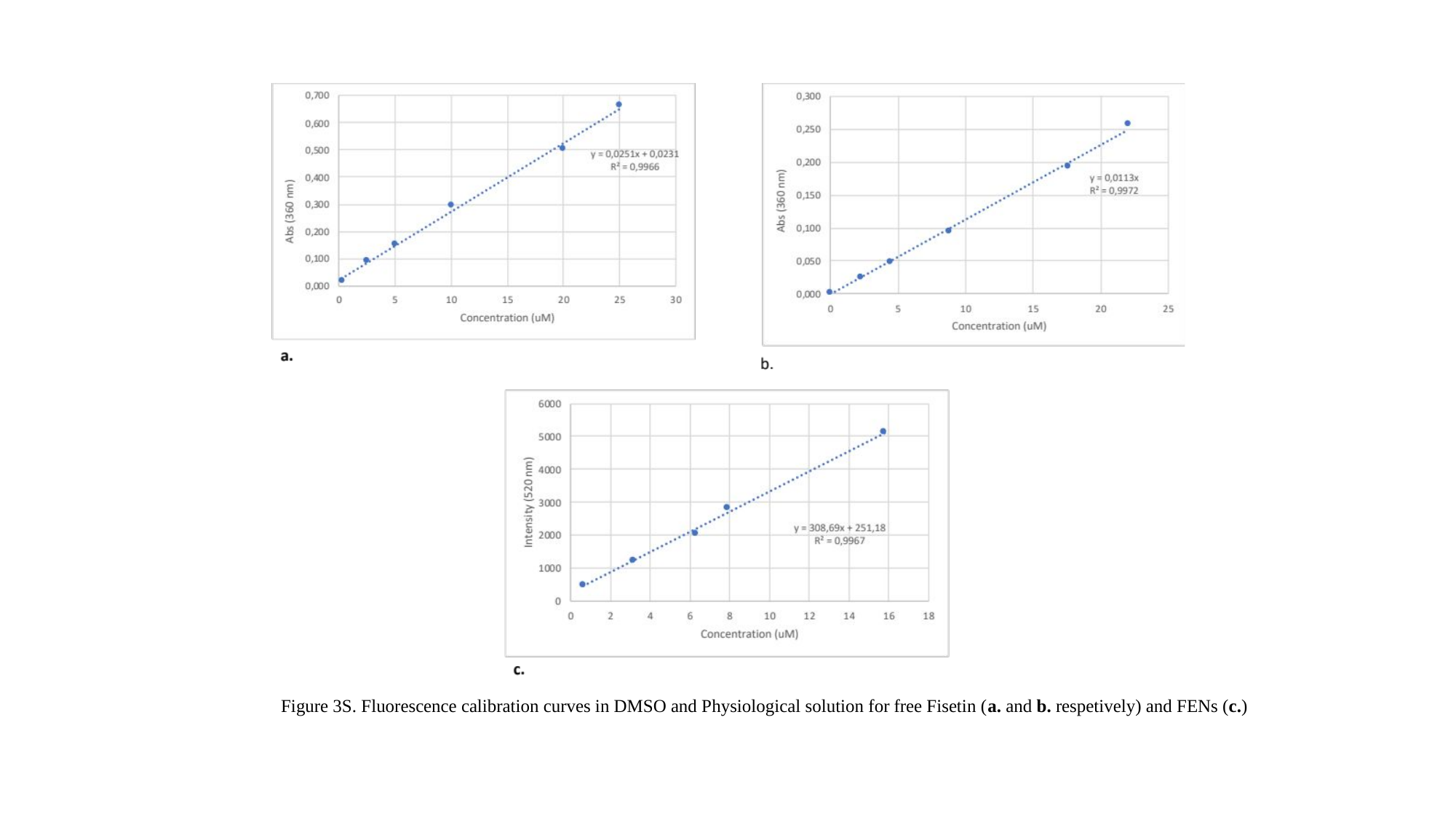

Figure 3S. Fluorescence calibration curves in DMSO and Physiological solution for free Fisetin (a. and b. respetively) and FENs (c.)
